# Supplementary material for: Wildfires disproportionately affected jaguars in the Pantanal
Source: Commun Biol. 2022 Oct 13;5:1028. doi: 10.1038/s42003-022-03937-1 (PMC9561719; doi:10.1038/s42003-022-03937-1)
Supplement: Supplementary file 2 — Supplementary Material [file 42003_2022_3937_MOESM2_ESM.pdf]

Supplementary Information

Wildfires disproportionately affected jaguars in the Pantanal

Alan Eduardo de Barros et al.

Supplementary Note 1

Complementary analysis (Results)

Real-time fire occurrence in jaguar home ranges (HRs)

In addition to the analyses considering the impacts of fire on HRs across multiple years, we analysed real-time fire impacts on jaguar home ranges (HR) (Figs.S10-S15). This analysis considered fires that occurred within jaguar HRs only during the individual monitoring period (2008–2016 in Brazil and 2005, 2006, and 2010 in Paraguay/Bolivia; Figs.S10-S16). Our real-time investigation allowed us to find that the effects of fire were negligible in the area used by the four non-resident individuals (Fig.S11). While this might be just an anecdotal observation (given the small sample size), it may also be that fires caused more impacts on high-quality habitats within the HRs of the resident jaguars.

Relationship of the year and project region with fire occurrence

We used a two-way ANOVA to evaluate the effect of year, project region, and ID on real-time fire occurrence within jaguar HRs (during real-time monitoring) and jaguar HRs (HRs compared across all years). The interaction between year and project region on fire occurrence was the most plausible model, since it generated the smallest residuals (Table S4). However, a major limitation in the real-time analysis was the unequal distribution of jaguar HRs in space and time. Years without monitoring (2007, 2017–2020) or with sparse or clustered monitoring (2005–2006, 2008–2009, and 2016) seemed to neglect or underestimate the impact of fires on the overall jaguar HRs (Figs. S12-S15). Conversely, the main approach used here (using 48 home ranges of resident jaguars across multiple years Figs.2 and 3 in the main text) was more representative in space and time.

Additional details about the cumulative impacts in the UPRB and Pantanal (Discussion)

An assessment of the integrity risk of aquatic ecosystems identified hydroelectric power plants, urbanization, and agribusiness as the top three stressors among 13 anthropic activities impacting the Upper Paraguay River Basin (UPBR)[1]. Environmental degradation (e.g., deforestation, erosion, sewage), economic activities (e.g., agriculture, mining), and infrastructure (e.g., dams, hydroelectric power plants, waterways, gas pipelines) are associated with the demand and pressure of an increasing human population in the UPRB. These environmental damages can interfere with drainage dynamics, flood pulses, and drought extent and, consequently, impact ecological richness, biodiversity, and ecosystem services [1-6].

The Pantanal occupies 38% of the UPRB[7], and the water that drains into the lowlands and floods in the Pantanal comes from springs in the highlands[6,8]. Thus, the removal of the vegetation cover that protects these springs in the neighbouring biomes within the UPRB (Cerrado, Amazonia, Atlantic Forest, and Chaco)[9] impacts the drainage, water quality, and hydrological regime of the Pantanal[1,4-7]. Deforestation, mining activities[2,10] and hydroelectric power plants (total ≈ 180, 47 of them installed or in operation and 133 planned)[11] change water quality and flow, causing erosion, river sedimentation[2,9], and reduction of outflow (up to 100% in drier years) and nutrient transport to the Pantanal floodplains[12,13]. Additional problems include reduced light, which is causing a decline or disappearance of aquatic plants and photosynthetic algae[1], hypoxia and a consequent cascade effect, such as reduced food availability for fish[1] and bioaccumulation of toxic mercury[10,14].

The implementation of a waterway on the Paraguay River, hydroelectric power plants, railroads, and highways have long been debated in academia, legal courts, and by stakeholders[2,15-20]. Overwhelming evidence of negative impacts has barred or reduced the development of large enterprises, such as the construction of a 3,440 km^2 north-south waterway from Cáceres (Brazil) to Nueva Palmira (Uruguay) and large hydroelectric power plants[2,15-18]. However, a subterfuge often used by stakeholders in many new enterprises is to propose several small hydroelectric plants, ports, or relatively short canalization projects as low-impact alternatives, without considering their cumulative impacts[21]. In addition, the supposed small individual impact is often used as an excuse to limit the decision-making to a smaller group of stakeholders[16,22], the so-called "tyranny of small decisions"[16,23]. And the quality of many Environmental Impact assessments in Brazil lacks scientific rigor[24]. The impact of the cumulative effects of small ventures can be only avoided by adopting a holistic perspective in a process involving scientists with relevant subject matter expertise, planners, politicians, and other social actors[16,23] in in-depth studies, with impartial evaluation and discussions (e.g., using a Strategic (or Integrated) Environmental Assessments (SEA) or equivalent approaches[11,21,25-27]). Successful regulation based on laws[27] and updated SEAs for the Pantanal[1,11], through a transparent and plural engagement of civil society, could bring invaluable advances for conservation and provide science-based guidance to attain sustainability[2,11,28,29]. See Fig.4 in the main text.

Supplementary Figures

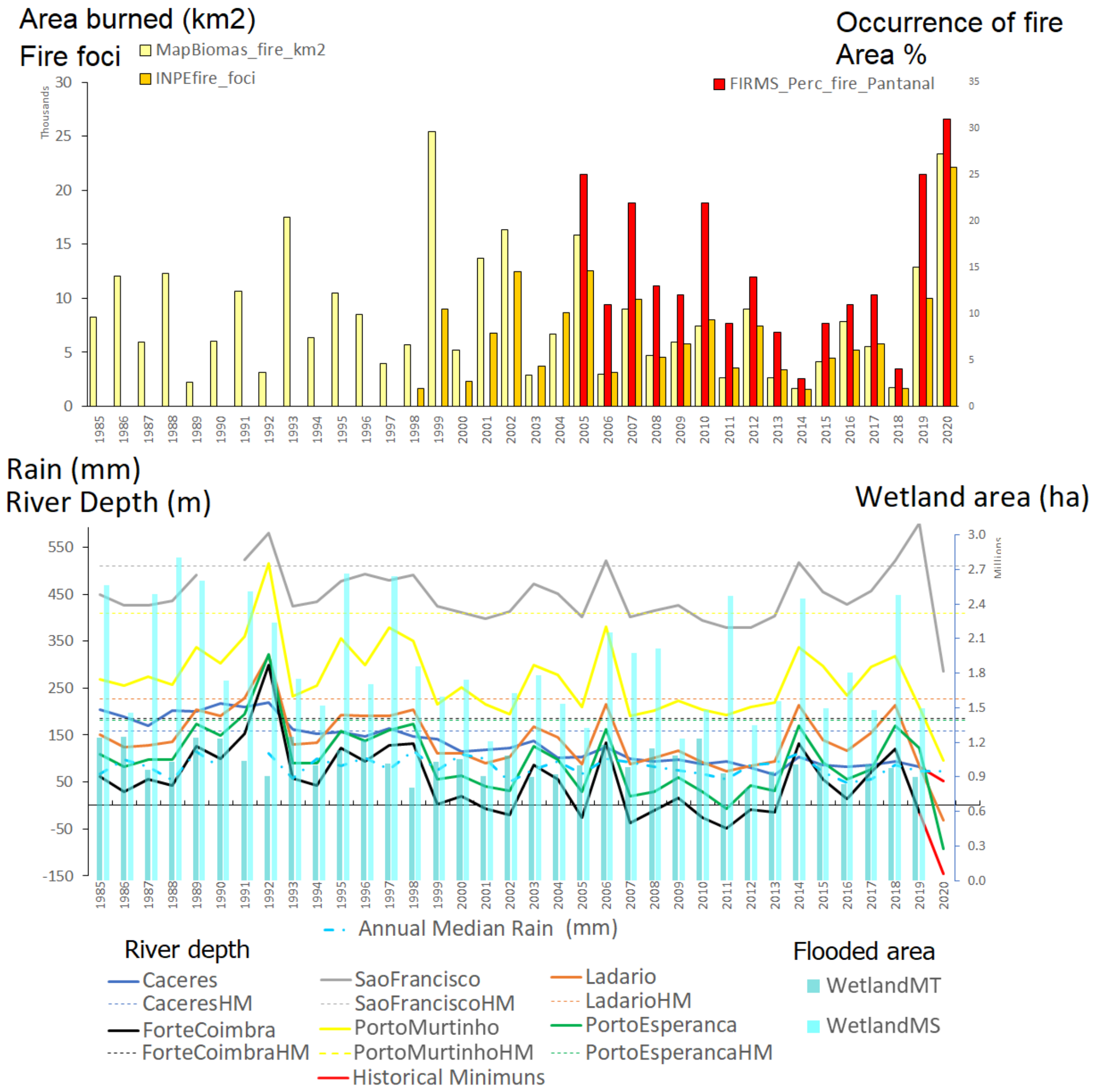

Fig.S1.*Top:* Comparative histograms of yearly fire available datasets, based on MODIS satellites indicating occurrence of fire foci (*INPEfire_foci*)[30], derived proportion of burned area (*FIRMS_Perc_fire_Pantanal*)[31,36] and burned area based on LANDSAT satellites (*MapBiomas_fire_*Km^2)[32]. *Bottom*: The continuous lines correspond to the minimum annual river depths (minimum quotas in meters from 1985 to 2020) at six Pantanal gauge stations in the Paraguay River. The dashed lines in the same colours correspond to the respective historical medians (HM) at each station[33,34]. The histograms correspond to the MapBiomas 5.0[35] annual wetland estimated area (ha) in the Pantanal areas of Mato Grosso (MT) and Mato Grosso do Sul (MS) from 1985 to 2019. The dashed-dotted blue line corresponds to the median annual rainfall (1967 to 2019).

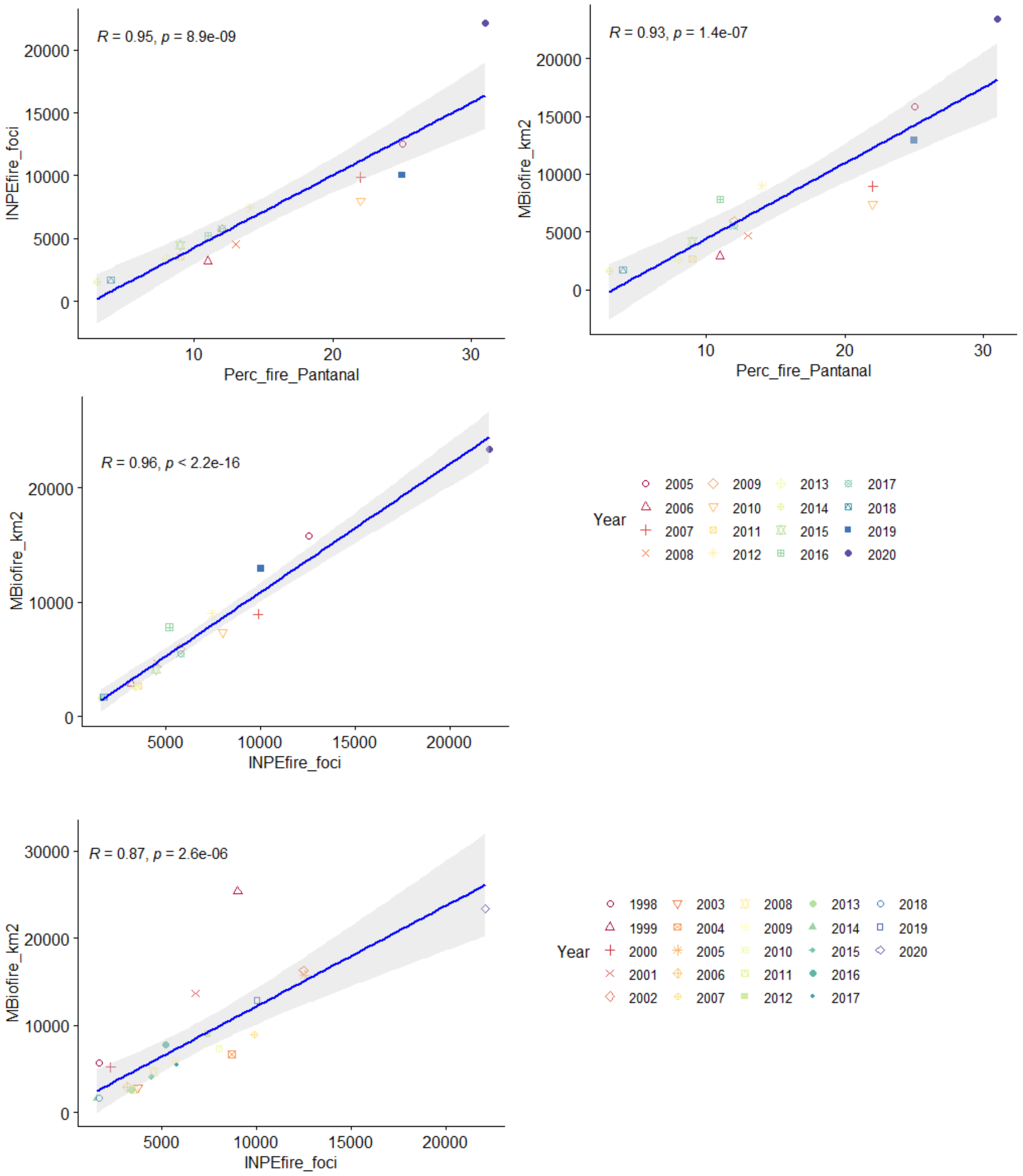

Fig.S2. Spearman correlation of yearly fire data. ***Upper panel:*** Correlation between the proportion of burned area estimated using FIRMS[31,36] fire occurrence dataset (*Perc_fire_Pantanal*) with occurrence of fire foci (*INPEfire_foci*)[30] and with burned area based on LANDSAT satellites (*MapBiomas_fire_*Km^2)[32] for the period between 2005-2020. ***Middle panel:*** Correlation between the (*INPEfire_foci*)[30] and (*MapBiomas_fire_*Km^2)[32] for the period between 2005-2020. ***Bottom panel:*** Correlation between the (*INPEfire_foci*)[30] and (*MapBiomas_fire_*Km^2)[32] for the period between 1998-2020.

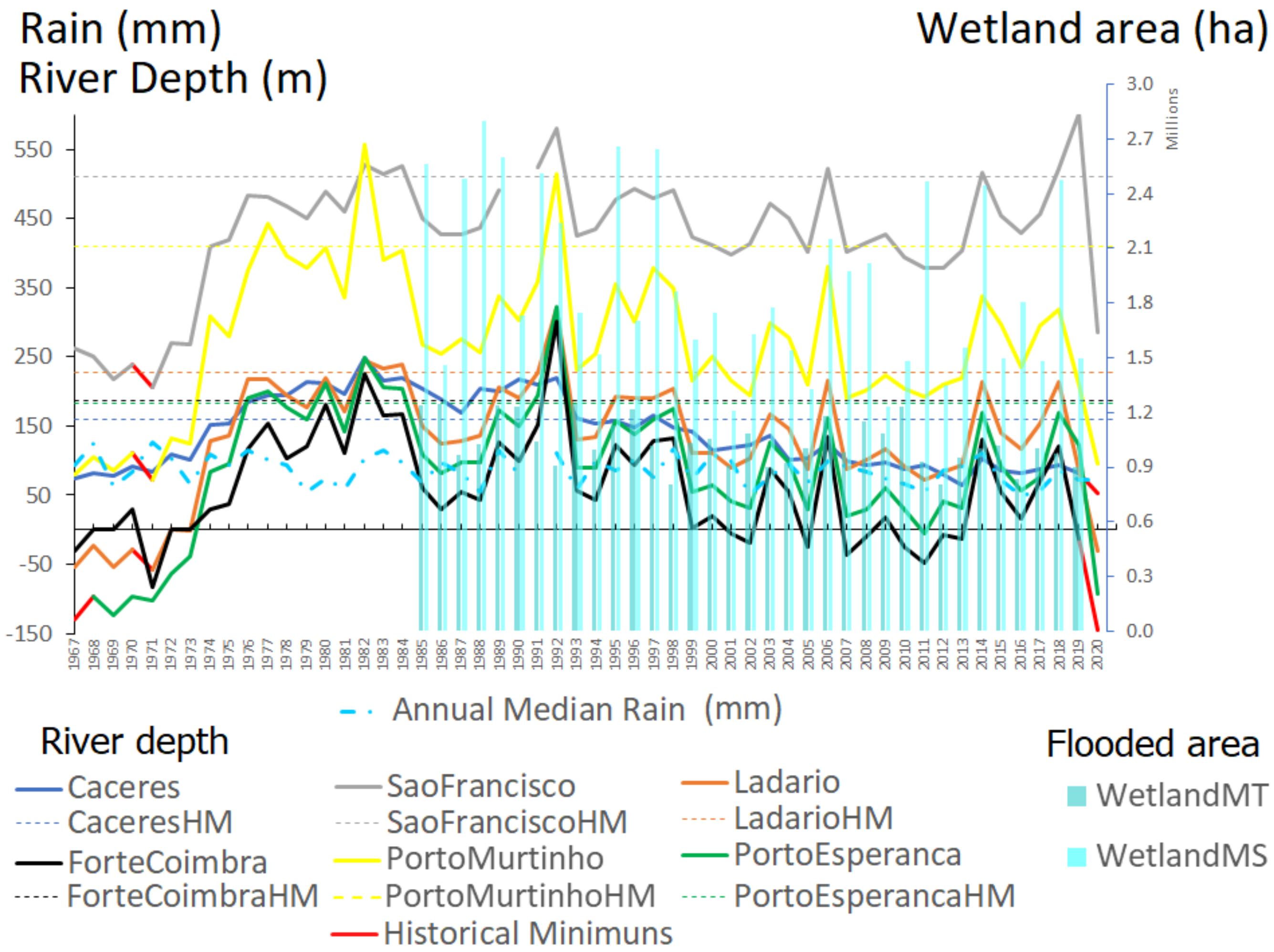

Fig.S3. The continuous lines correspond to the minimum annual river depths (minimum quotas in meters from 1967 to 2020) at six Pantanal gauge stations in the Paraguay River. The dashed lines in the same colours correspond to the respective historical medians (HM) at each station[33,34]. The histograms correspond to the MapBiomas 5.0[35] annual wetland estimated area (ha) in the Pantanal areas of Mato Grosso (MT) and Mato Grosso do Sul (MS) from 1985 to 2019. The dashed-dotted blue line corresponds to the median annual rainfall (1967 to 2019).

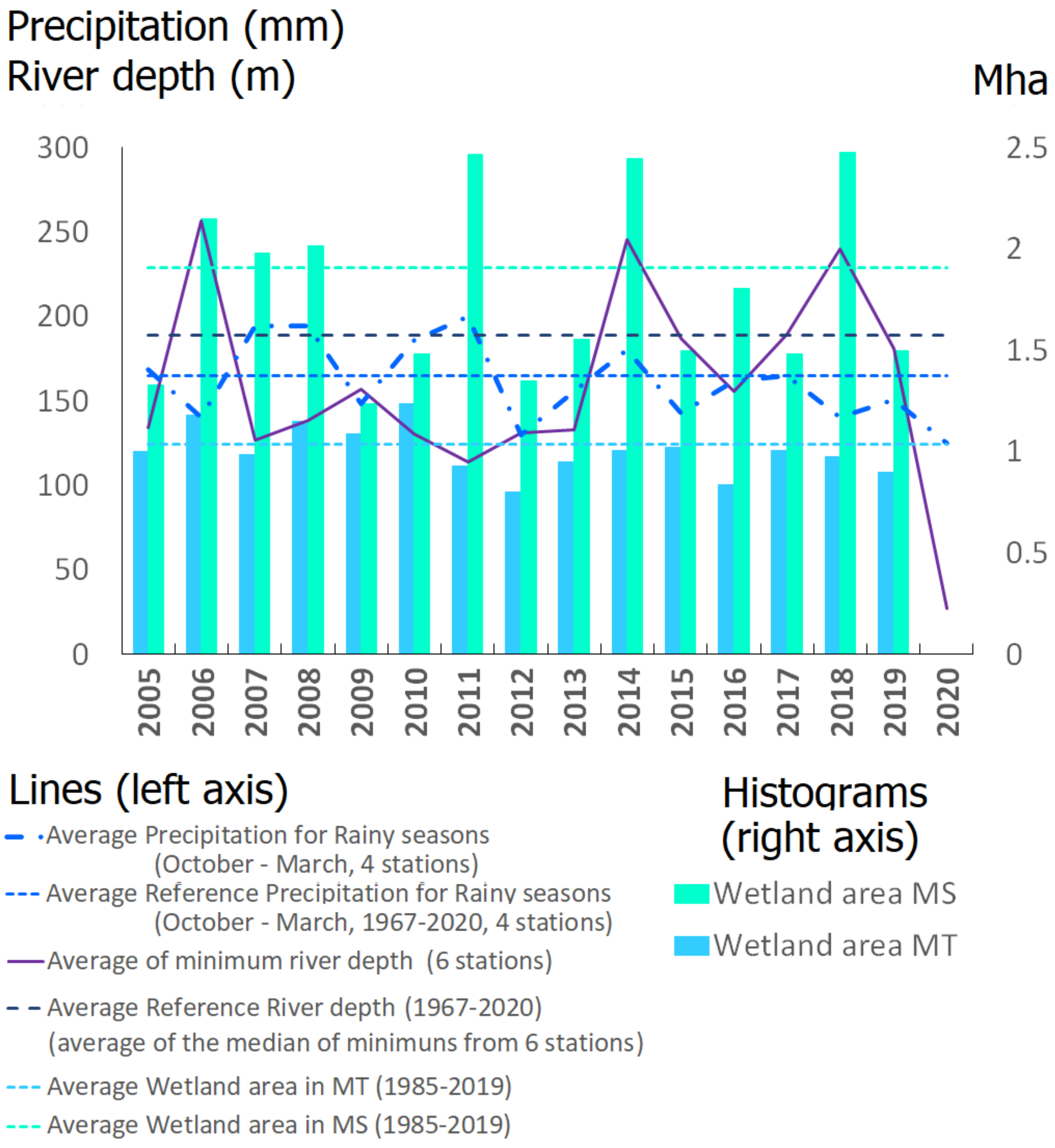

Fig.S4. Hydrological conditions during the jaguar monitoring period (2005–2016) and in subsequent years. The continuous lines correspond to minimum annual river depths (minimum quotas in meters from 2005 to 2020) at six Pantanal gauge stations in the Paraguay River[33,34], and the dashed lines with the same colours correspond to the respective averaged references (average, from six stations, of medians of yearly minimums of each station during the monitoring period). The dashed-dotted line corresponds to median annual rainfall (reference 1967 to 2019)[37]. The histograms correspond to the MapBiomas 5.0[35] annual wetland estimated area (ha) in the Pantanal areas of Mato Grosso (MT) and Mato Grosso do Sul (MS) from 2005 to 2019. The dashed lines in the same colours correspond to the respective averages (1985 to 2019).

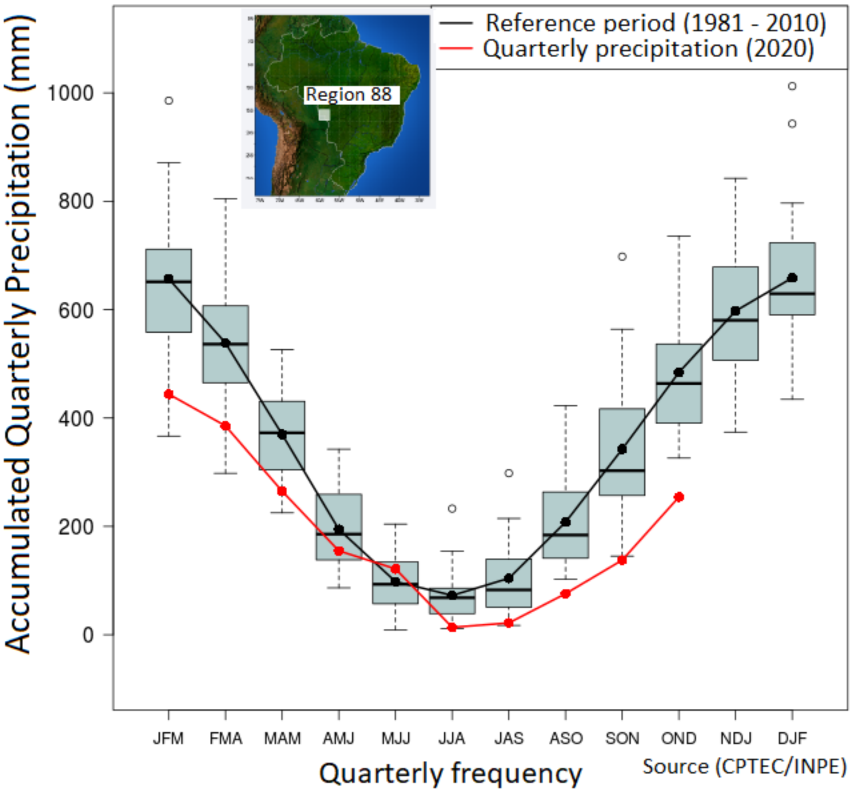

Fig.S5. Quarterly rainfall in 2020 in comparison with rainfall in the reference period (1981–2010)[38].

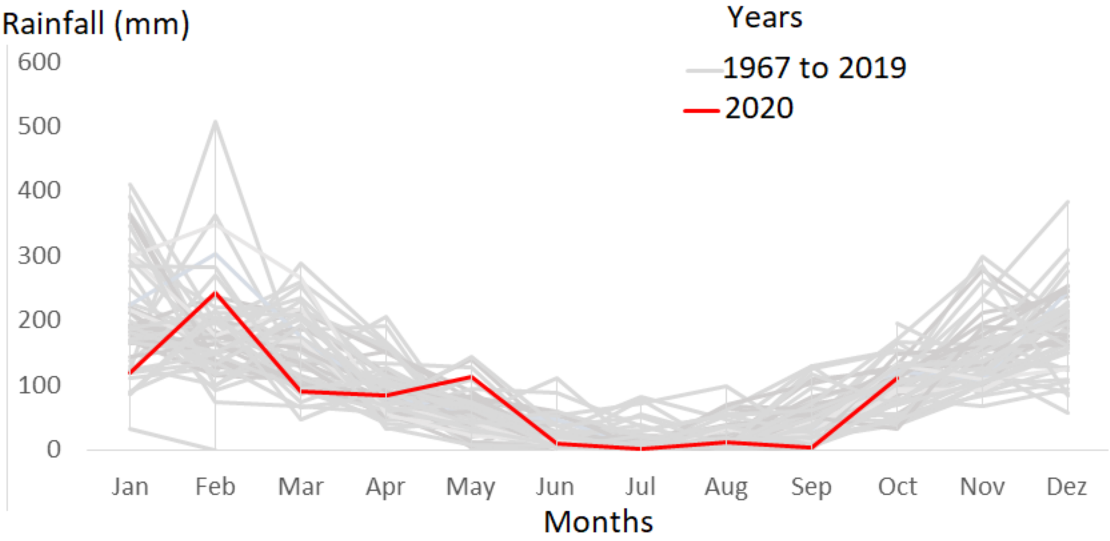

Fig.S6. Monthly rainfall per year (considering the median of 4 stations in the UPRB: Cáceres, Corumbá, Cuiabá, and Campo Grande)[37], with 2020 in red. Data acquired for 2020 was limited until October.

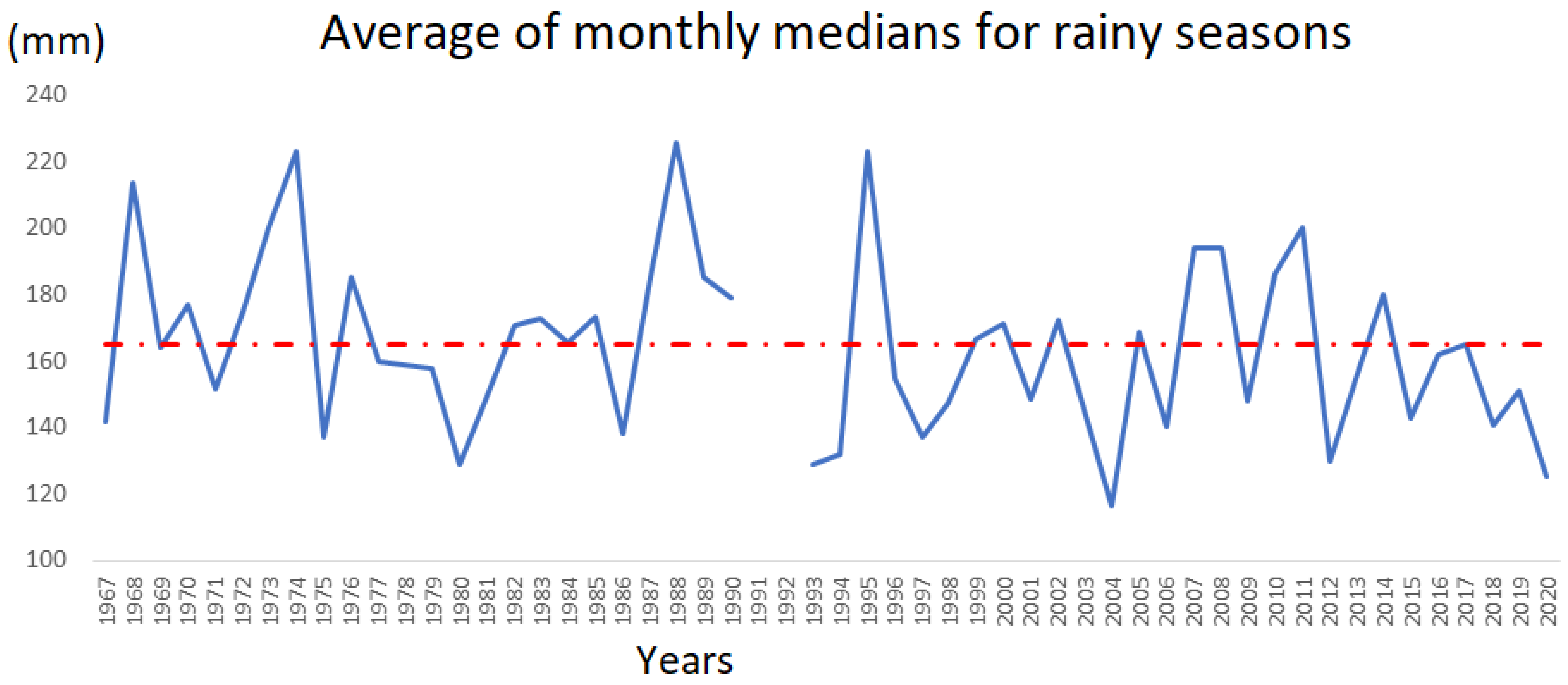

Fig.S7. Average monthly medians for the wet seasons (considering four stations in the UPRB: Cáceres, Corumbá, Cuiabá, and Campo Grande)[37]. Note that 28 years had estimates below the historical average, with five years (1980, 1993, 1994, 2004, 2012) having estimates more than 20% below the historical average[37]. Data acquired for 2020 was limited until October.

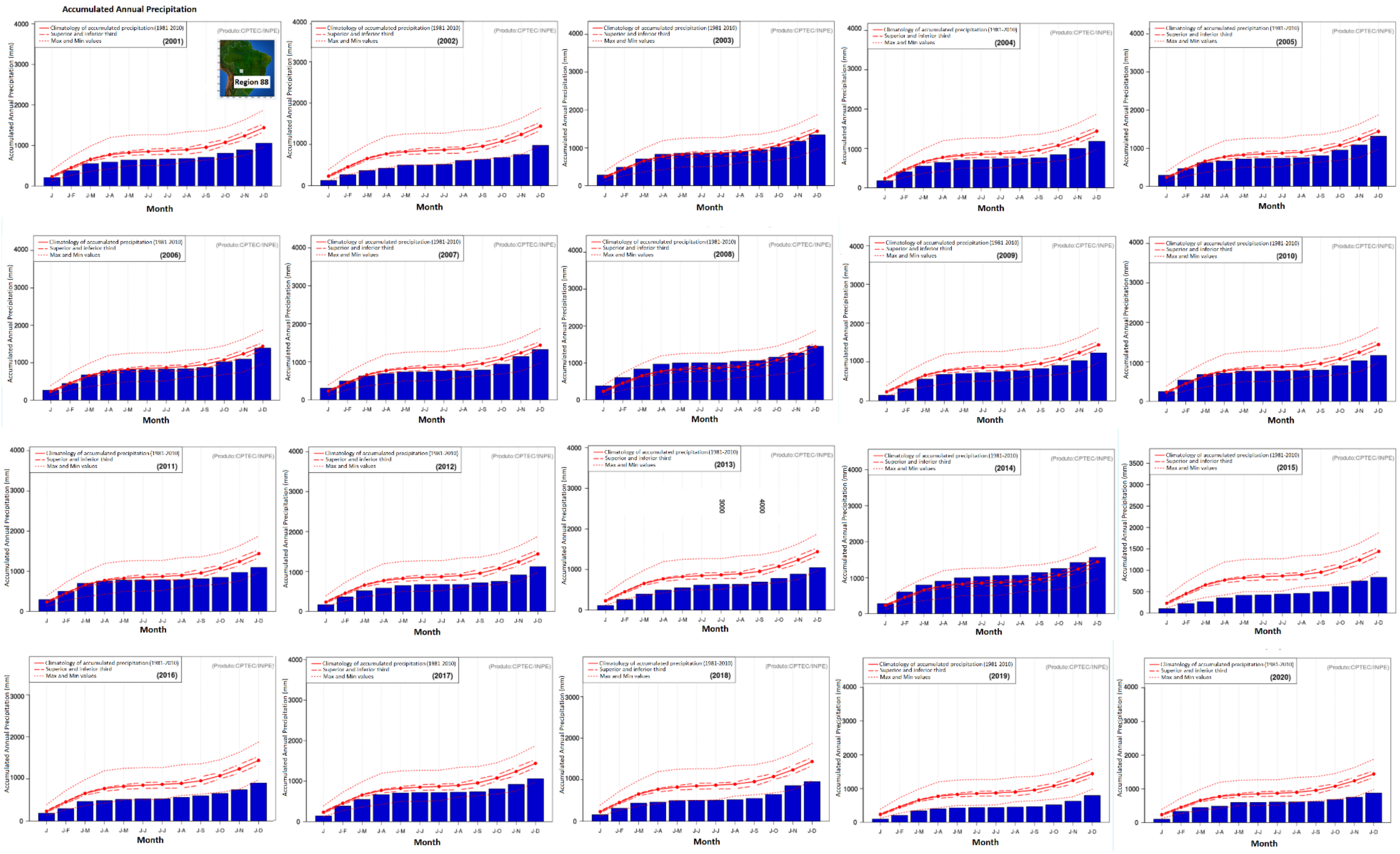

Fig.S8. Annual rainfall from 2001 to 2020 in the Pantanal. Note that after 2014, accumulated rainfall often falls below the minimums (reference period 1981–2010; translated and adapted from CPTEC/INPE)[38].

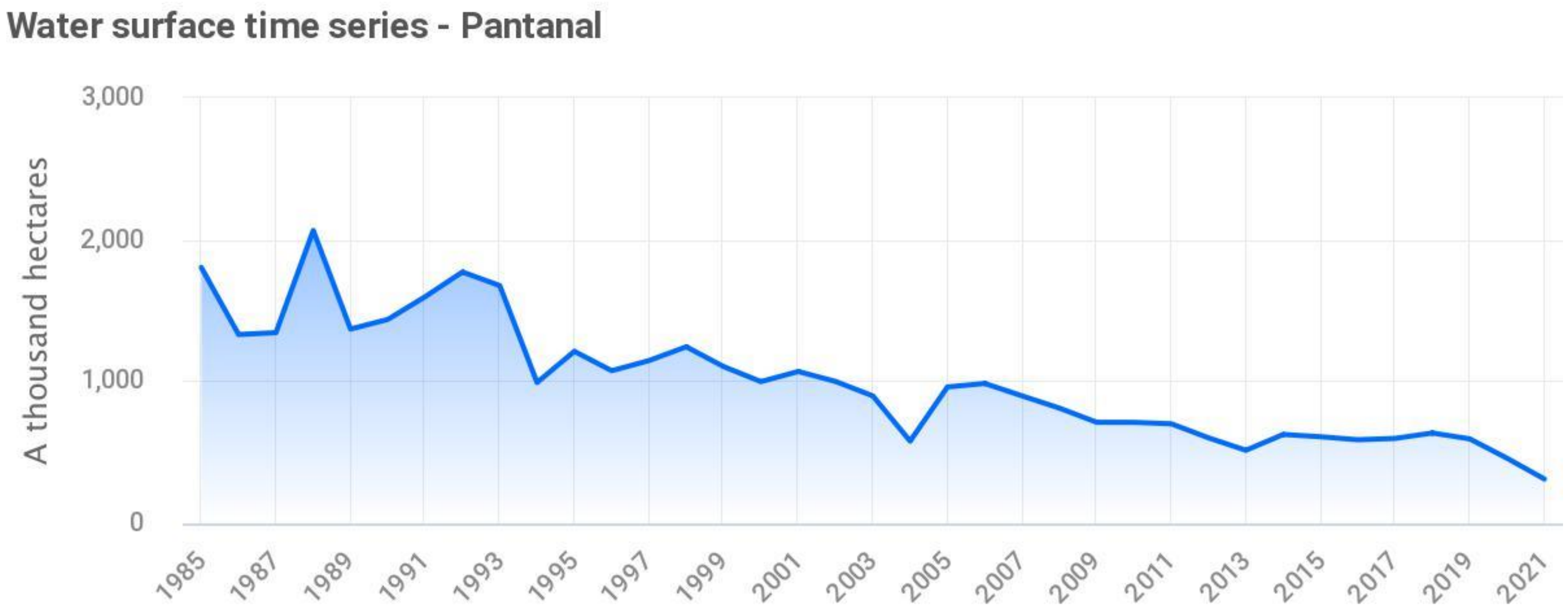

Fig.S9. Water surface time series from 1985 to 2021 in the Brazilian Pantanal (MapBiomas)[39].

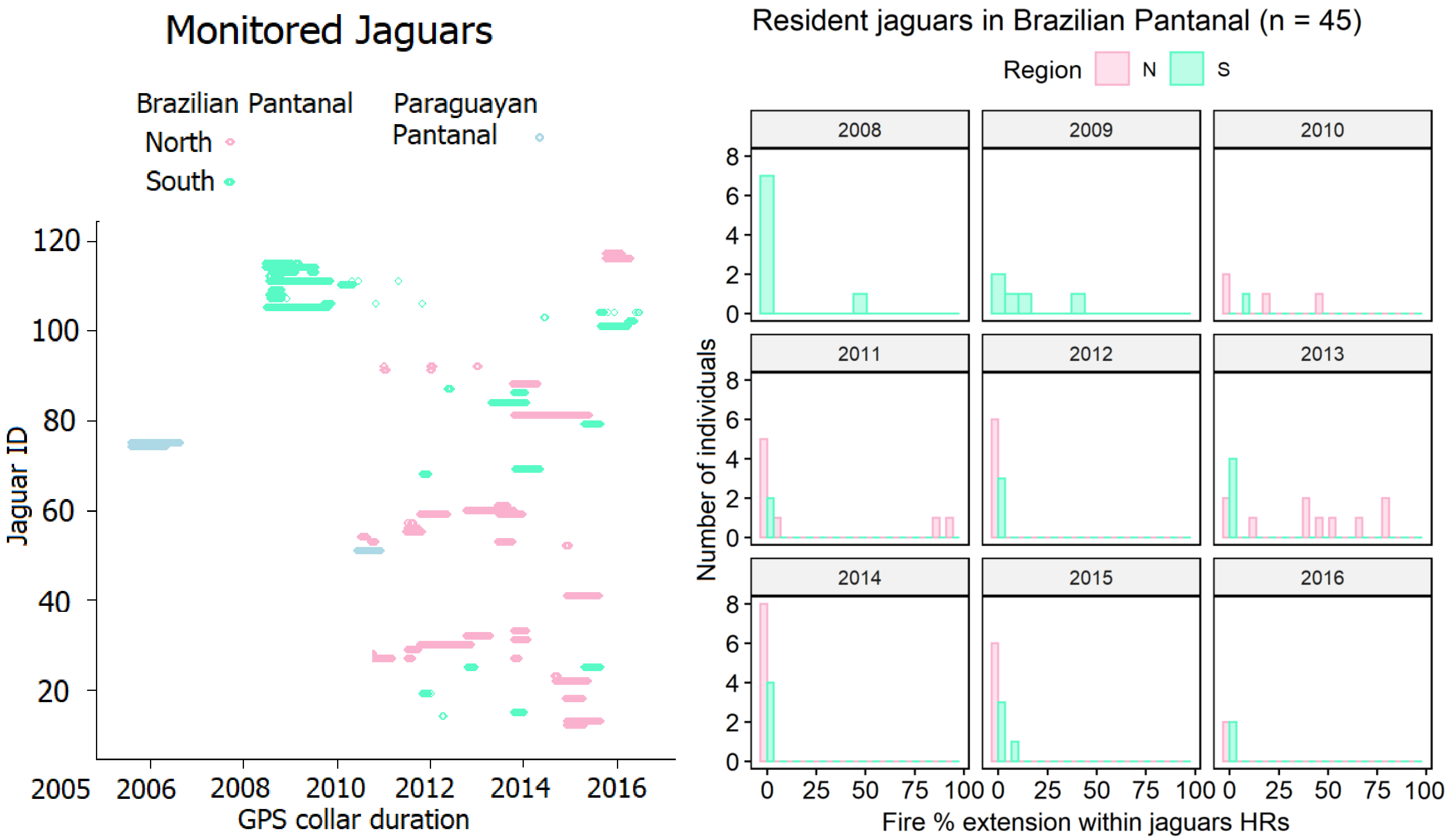

Fig.S10. The left panel shows the monitoring period for GPS-collared jaguars[40]. The right panel presents the yearly percentage of fire impacting jaguar HRs during the individual monitoring period in the Brazilian Pantanal.

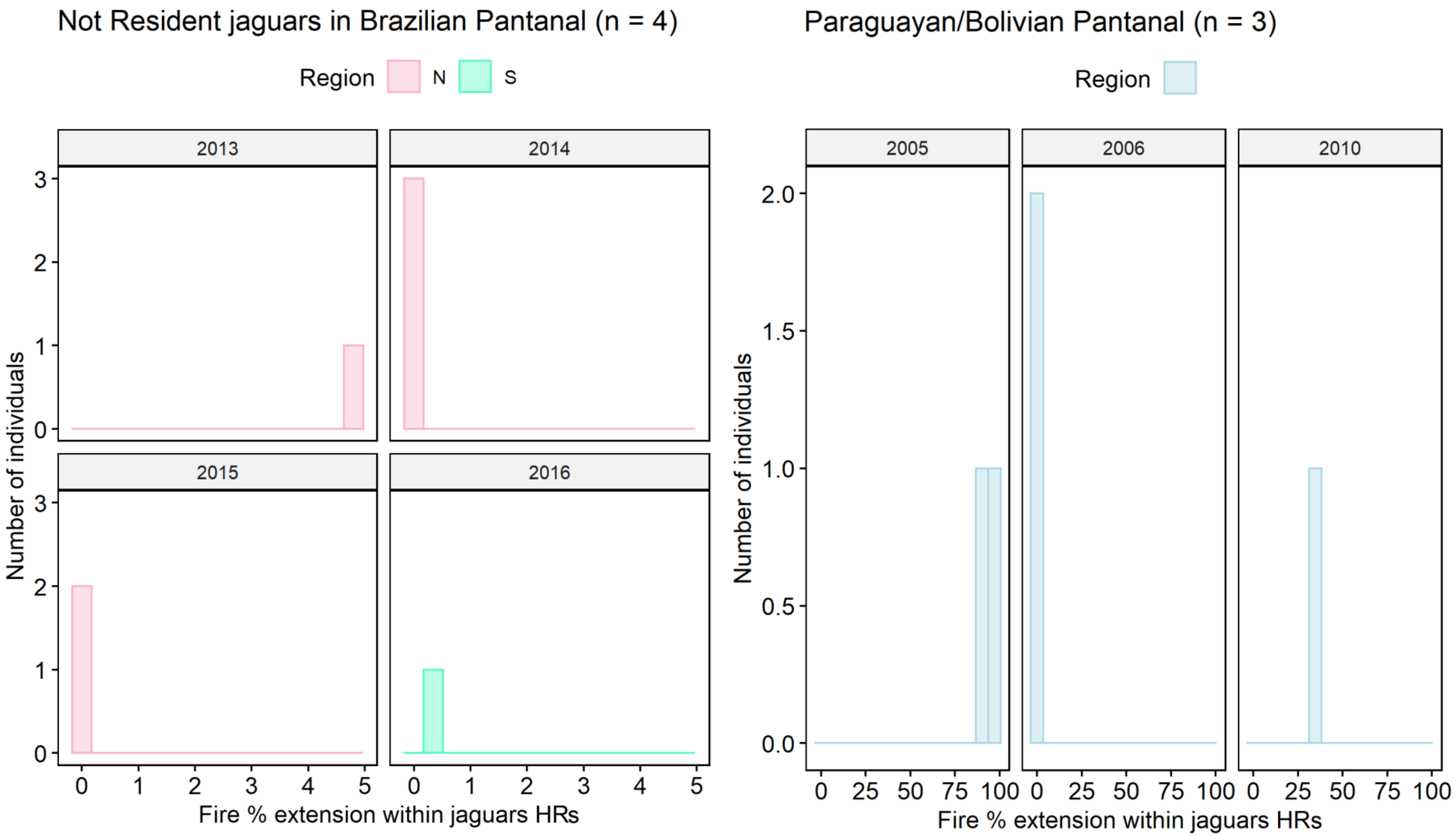

Fig.S11. Percentage of fire occurrence matching individual jaguar areas during the monitoring period. Non-resident jaguars in Brazil (left) and resident jaguars from Paraguay/Bolivia (right). Note the low fire occurrence within the areas used by non-resident jaguars.

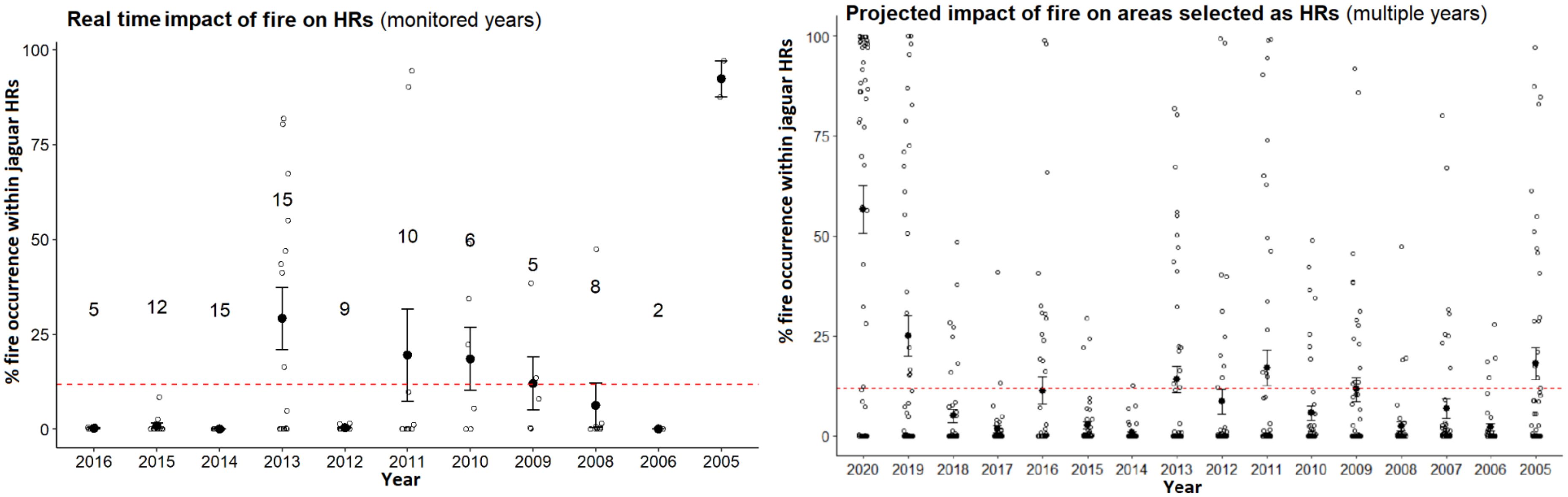

Fig.S12. **(Left)** Real-time impact with the percentage of fire occurrence matching individual jaguar areas during the GPS monitoring period. This plot includes 52 individuals from the Brazilian Pantanal (45 residents and 4 non-residents) and the Paraguayan Pantanal (3 residents). The numbers in the plot represent the number of individuals monitored each year. **(Right)** Projected impact of fire on areas selected as home ranges (HRs) of 48 resident individuals from 2005 to 2020. This projection allowed us to explore the impacts of fire on jaguar HRs for years in which tracking data were unavailable (as was the case for 2020) (see Table S4).

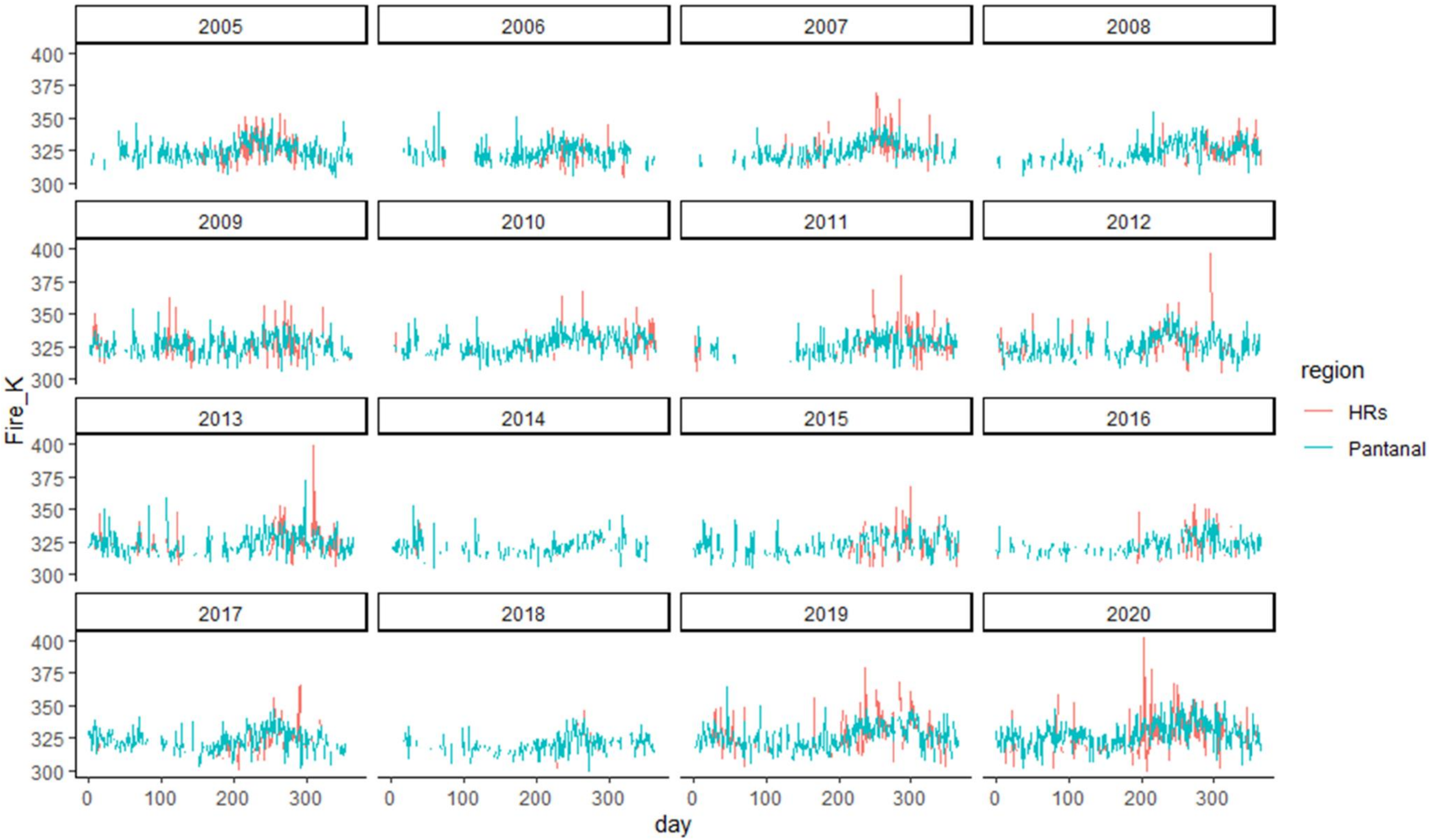

Fig.S13. Intensity of daily fire occurrence per year considering areas selected as HRs (stable jaguar priority areas) in **red**, and whole Pantanal in **blue** for the period of 2005–2020.

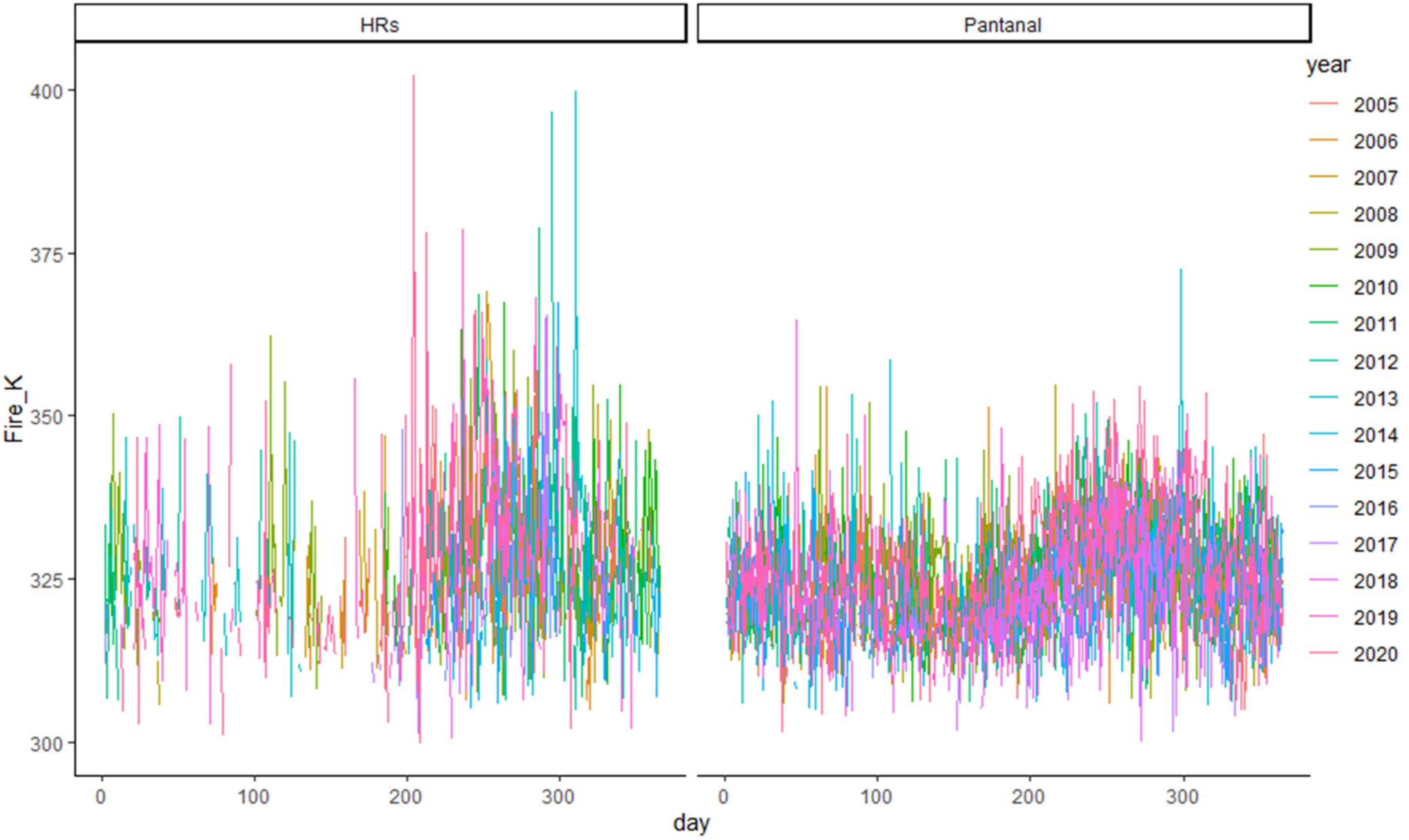

Fig.S14. Intensity of daily fire occurrence per year considering areas selected as HRs and whole Pantanal for the period of 2005–2020.

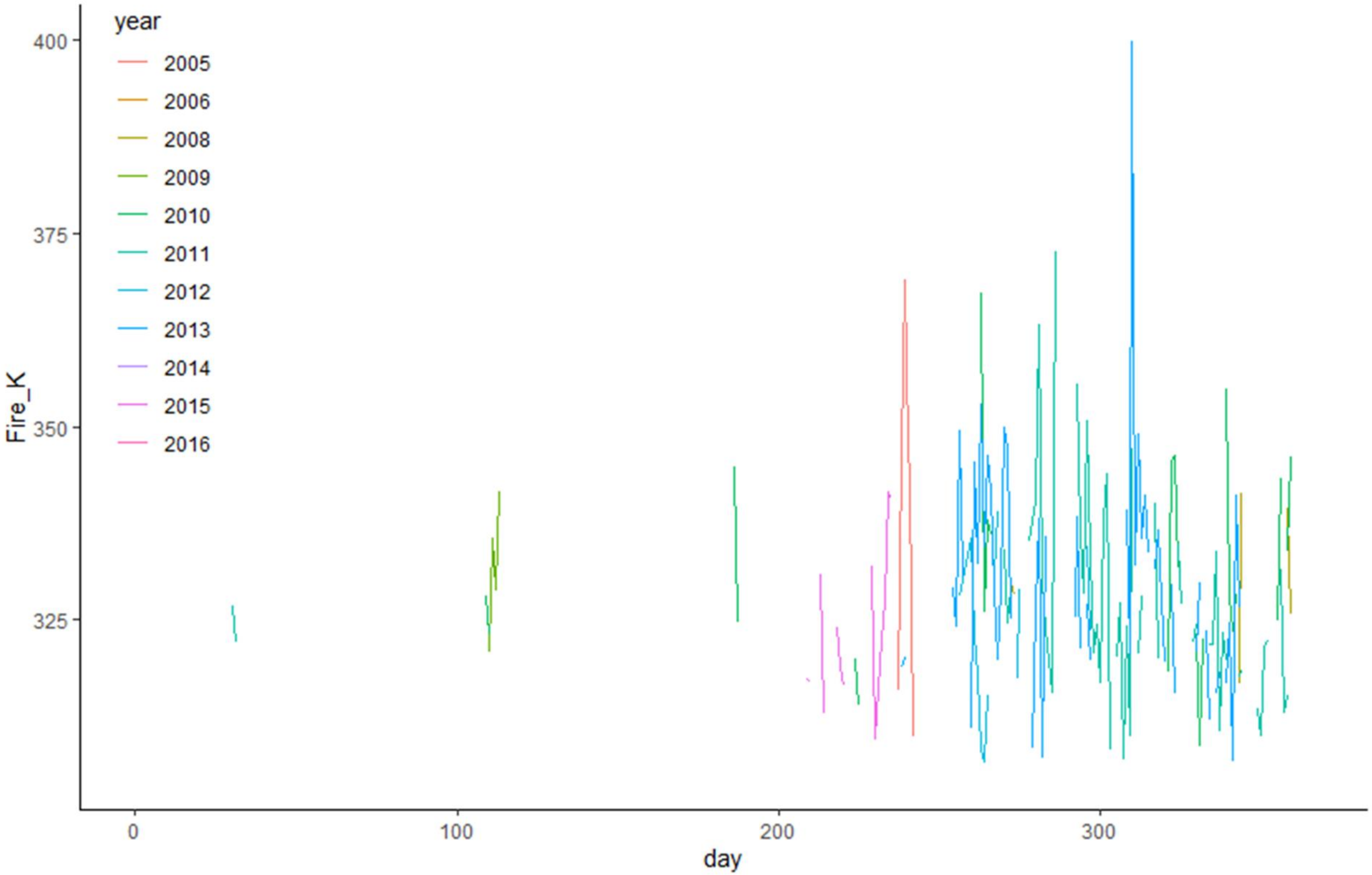

Fig.S15. Intensity of daily fire occurrence per year coinciding with HRs during the real time monitoring period of individuals.

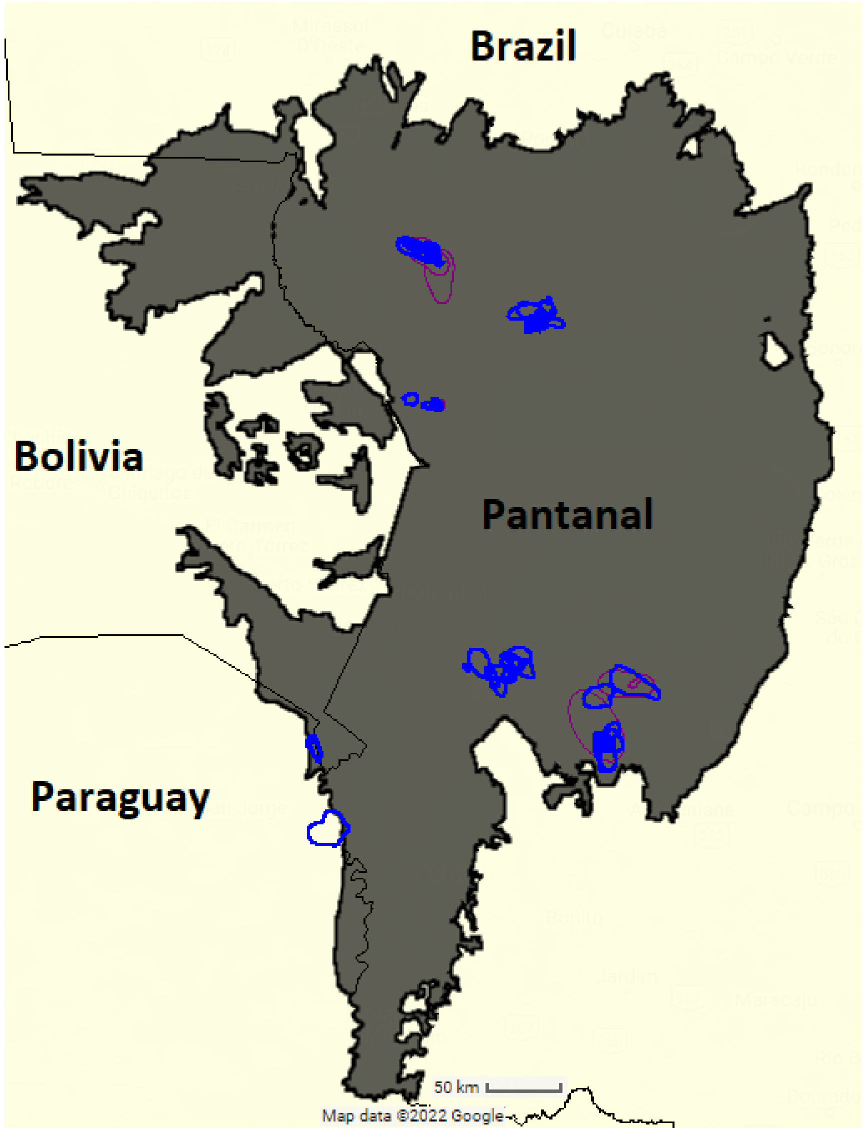

Fig.S16. Resident jaguars (blue)[40] from the Brazilian and Paraguayan/Bolivian Pantanal and non-residents jaguars (purple)[40]. Map boundaries data sources[52-54] acquired through Google Earth Engine[41].

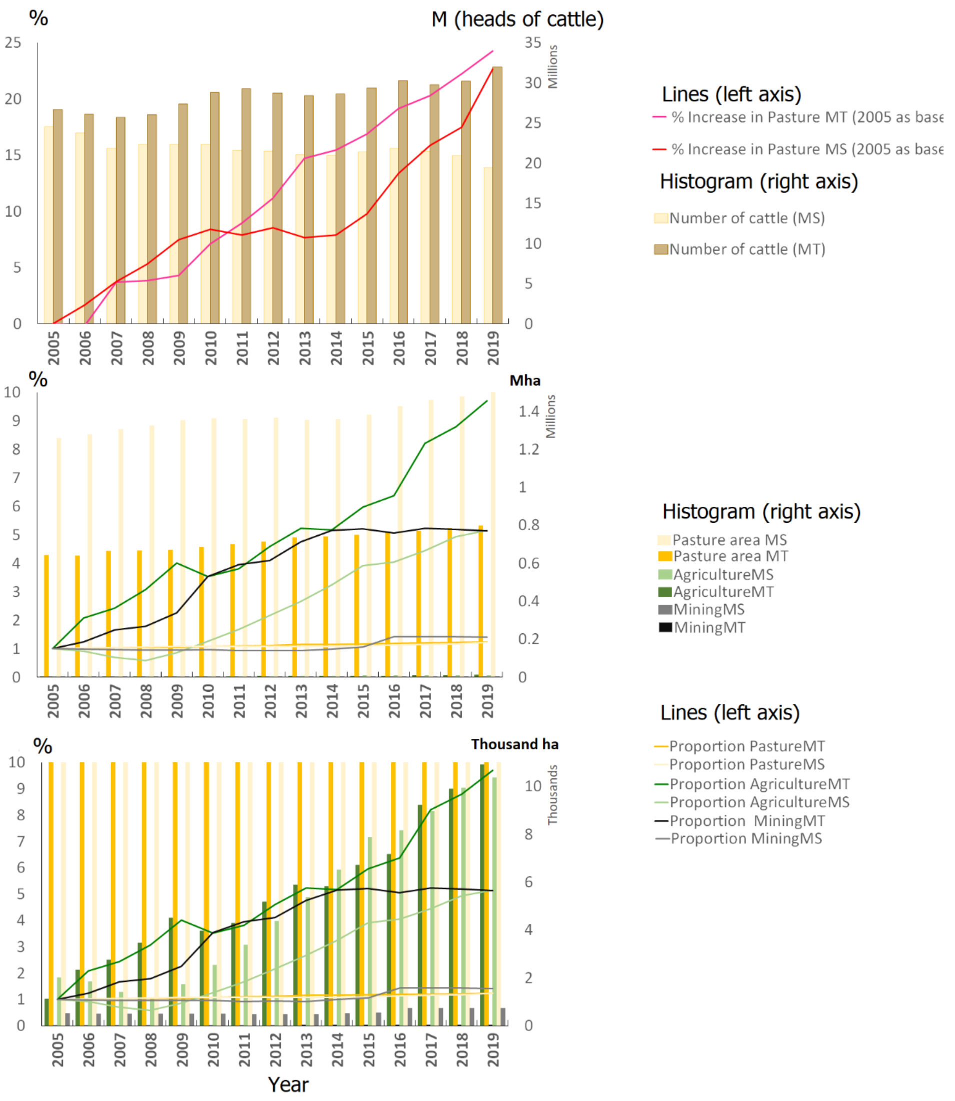

Fig.S17. Comparison of the main anthropic activities in the Brazilian Pantanal, states of Mato Grosso (MT) and Mato Grosso do Sul (MS)[35,42]. Cattle livestock is the most abundant economic activity. It continues to increase in Pantanal areas of both states, although the number of cattle and pastures in the Cerrado has decreased (see Fig.S18). Nonetheless, agriculture was the anthropic activity with the highest percentage growth (MapBiomas data)[35].

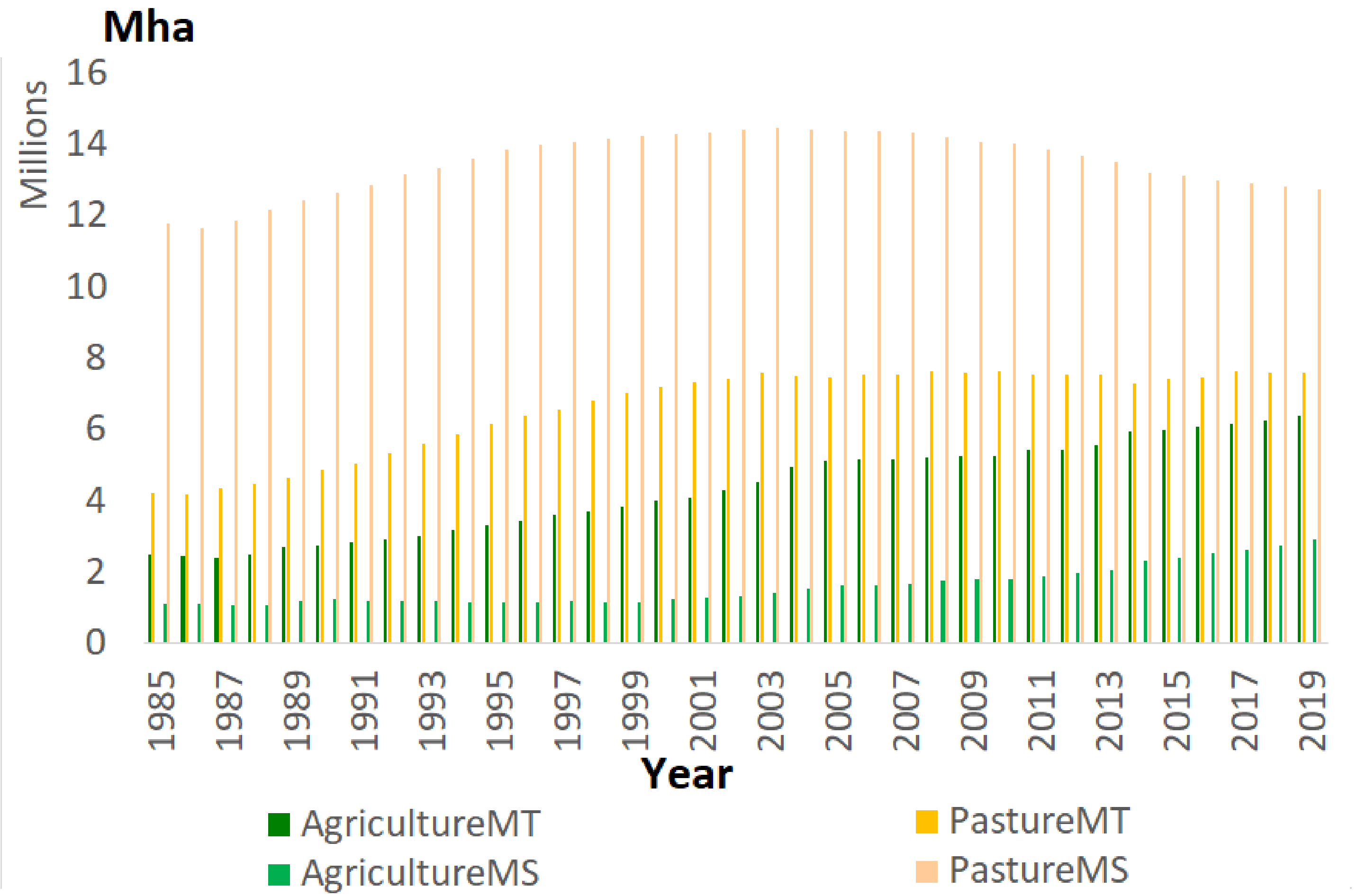

Fig.S18. Main anthropic activities in the Brazilian Cerrado, states of Mato Grosso (MT) and Mato Grosso do Sul (MS) (MapBiomas data[35]). Cattle livestock is still the most abundant economic activity, but the area occupied by agriculture (particularly soybean plantations) is increasing[35].

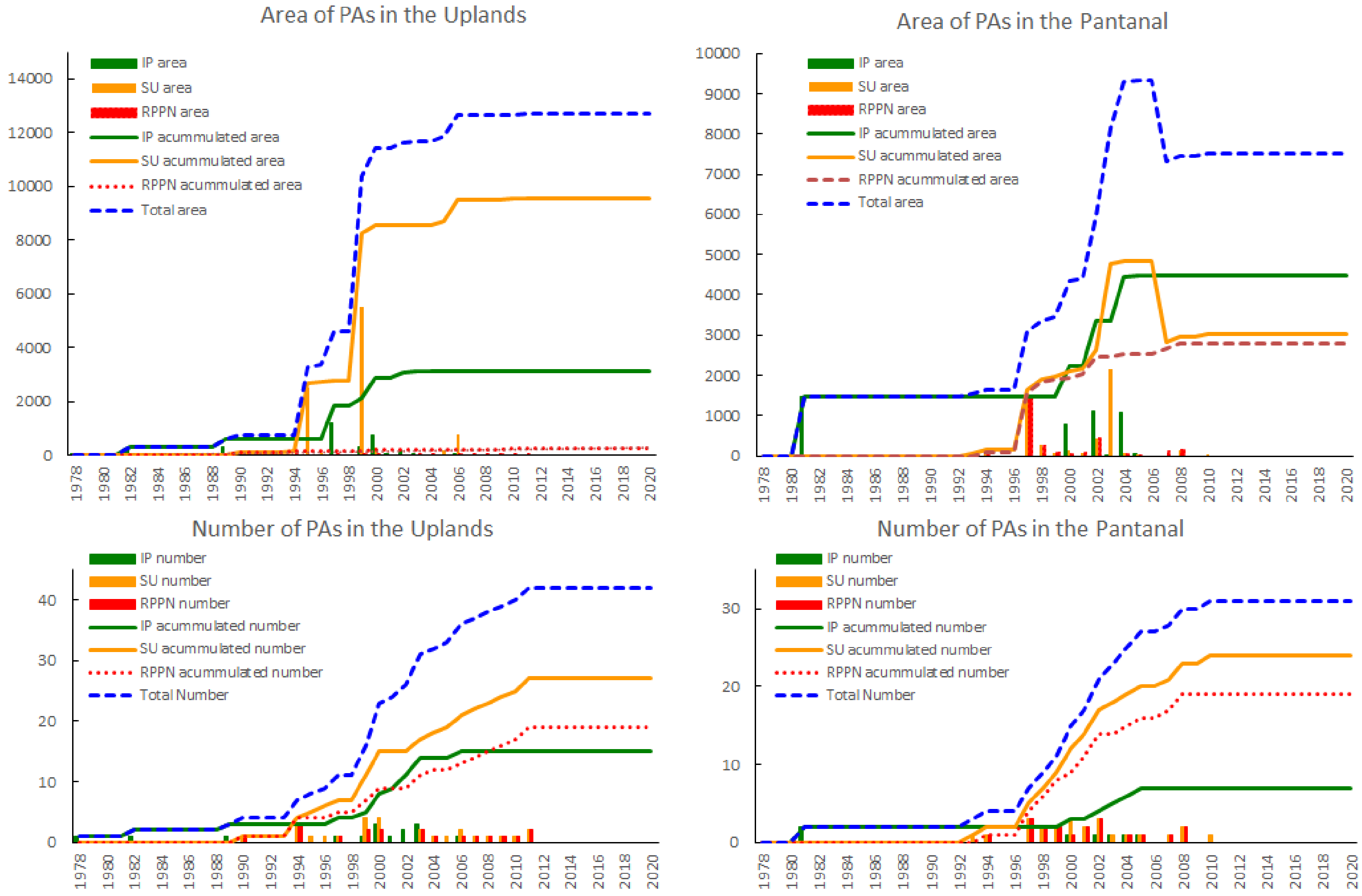

Fig.S19. Creation and maintenance of protected areas in the Brazilian Pantanal and the surrounding Uplands within the Brazilian Upper Paraguay River Basin. The upper panels show the accumulated area. The bottom panel show the accumulated numbers. IP = PAs of Integral Protection. SU = PAs of Sustainable Use. RPPNs = Private Reserves. SU = PAs that include RPPNs, Park Roads, and other categories of sustainable use. RPPNs are shown in detail, considering that almost 95% of the Pantanal are on private land. IP areas and RPPNs form most of the PAs in the Pantanal but are a minority in the Uplands. The areas have remained stable since the creation of the last PAs in the Uplands in 2011 and since the revocation of the Environmental Protected Area of Pontal dos Rios Itiquira and Correntes in 2006, reducing the total of PAs in Pantanal by almost 20%. Adapted from Tomas et al. 2019[2] and Chaves & Silva 2018[43] using the following sources: MMA 2020[44], IMASUL 2019[45], ICMbio 2021[46], ICMbio - SIMRPPN 2021[47]. Data used to make the charts are shown in Table S3.

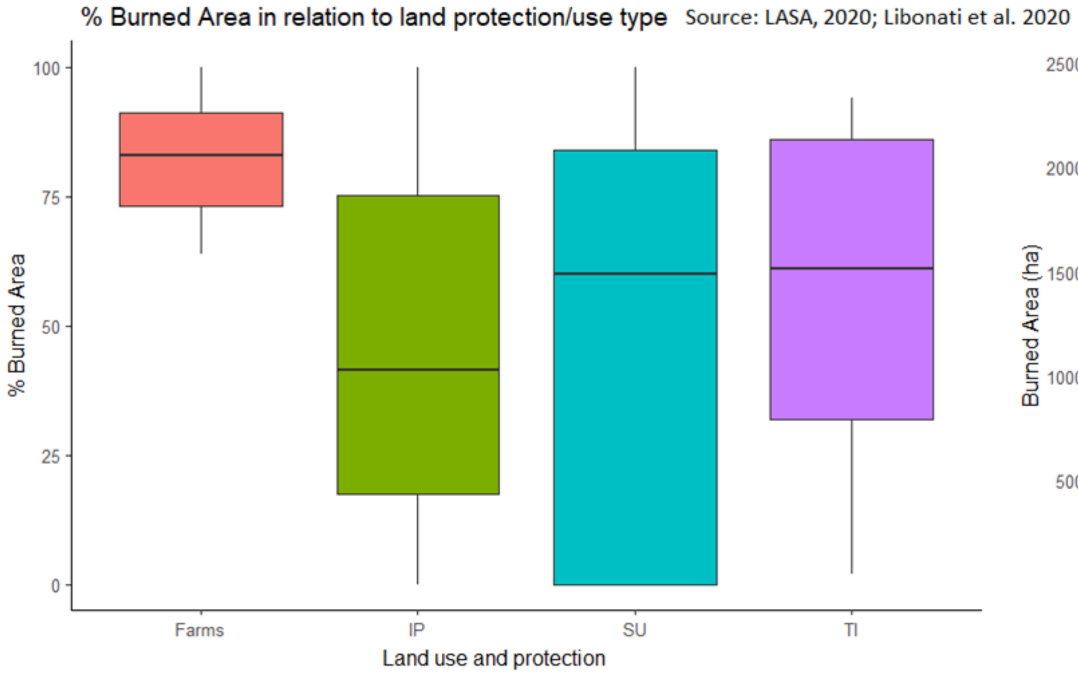

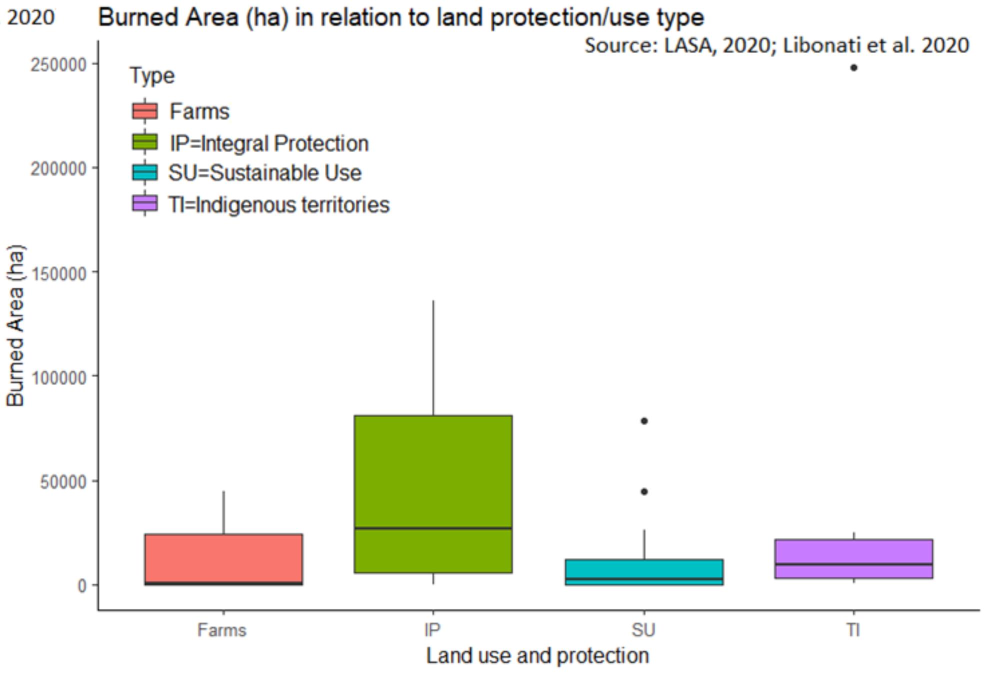

Fig.S20. Percentage variation (left) and burnt area (right) of the Brazilian Pantanal. Note that farms presented the highest medians for the percentage of burnt areas, while PAs of integral protection presented the highest absolute values of burnt areas. ANOVA applied to evaluate the effect of land protection/use type categories on the percentage and amount of burnt areas found no significant difference ($p > 0.05$) among the category types. Reference sources: LASA[48], Libonati et al.[49]. Data used in the analyses are shown in Table S5.

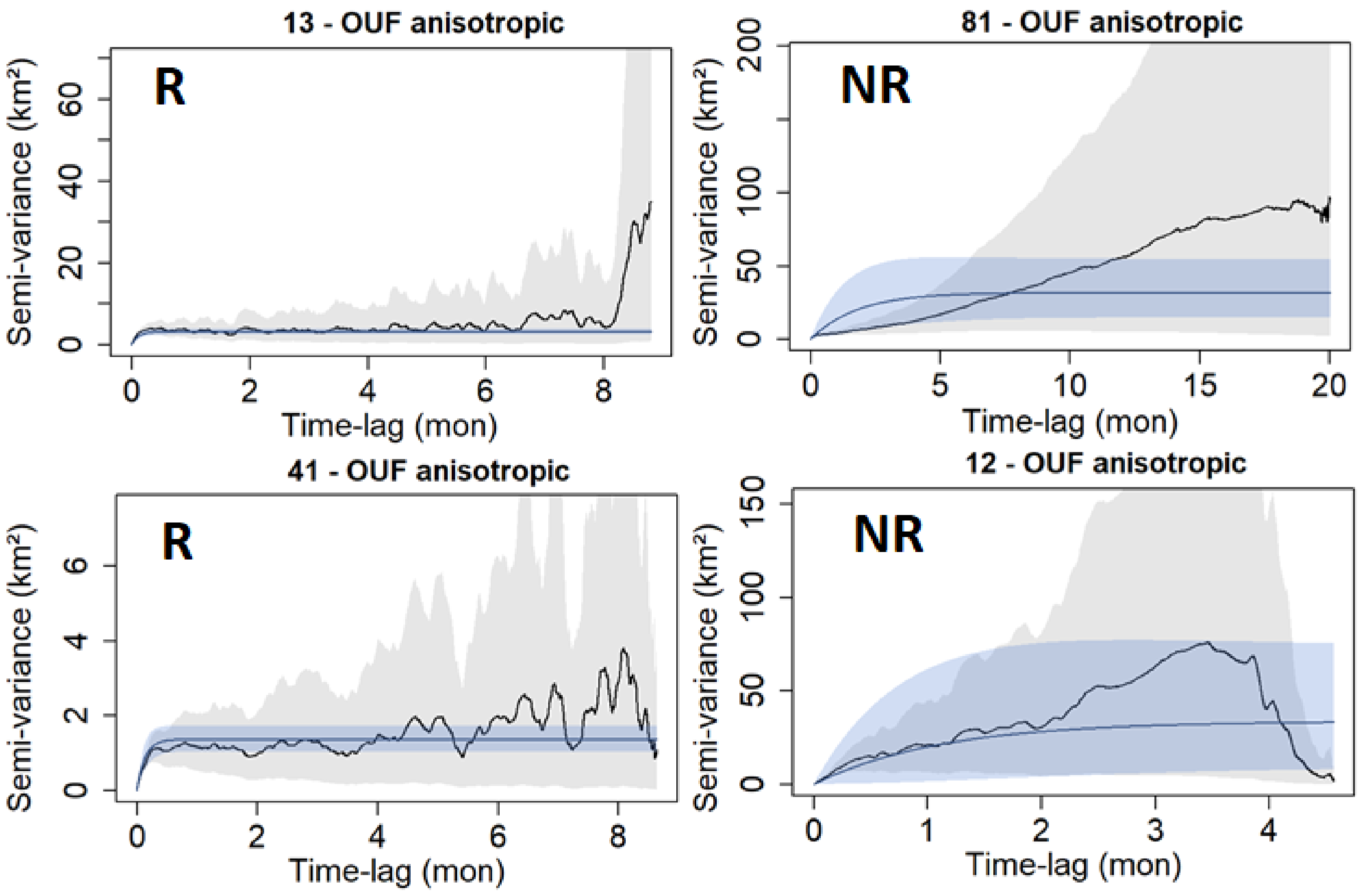

Fig.S21. Variograms of two resident (R, left) and two non-resident (NR, right) jaguars from the Pantanal. The best-fit models are represented by the blue line and their 95% Cis[50,51]. Non-resident jaguars lack a clear asymptote despite the long monitoring time, also reflected by a low number of range crossings. Numbers at the top are individual identifiers. OUF anisotropic corresponds to the best-fit model (Ornstein-Uhlenbeck-F), capturing autocorrelated locations and velocities for all R and NR jaguars shown below (see Table S6).

Supplementary Tables

Tab.S1. Areas and percentages of Brazilian protected areas (PAs) in the Pantanal, Uplands, and within Paraguay River Basin (UPRB). Brazilian UPRB corresponds to the basin area in Brazil. Total UPRB corresponds to the total basin area (multiple countries), and Total PRB corresponds to the entire Paraguay River Basin (PRB).

		PAs in the Brazilian UPRB (2020)		
**REGION BOUNDARY AREA**	**km^2**	**PA area km^2**	**%**	**number**
**Brazilian Pantanal**	150355	7506	5.0	31
**Brazilian Uplands**	212025	12684	6.0	42
**Brazilian UPRB**	362380	20190	5.6	73
**Total UPRB**	600000	20190	3.4	73
**Total PRB**	1100000	20190	1.8	73

Main sources: adapted from[2, 43-47], Brazilian Pantanal boundary (IBGE)[52].

Tab.S2. Comparative between Brazilian PAs in 2020 and the years in which the last area changes occurred. Note that PAs in the Brazilian Pantanal have decreased by almost 20% since 2007 and have remained the same in the Uplands since 2011.

**Type of PA**	**Pantanal**		**Uplands**	
**Year**	**2006**	**2020**	**2011**	**2020**
**Integral Protection km^2**	4491	**4491**	3140	**3140**
**Sustainable Use km^2**	4842	**3014**	9543	**9543**
**Total km^2**	9333	**7506**	12684	**12684**

Main sources: adapted from[2, 43-47].

Tab.S3. Protected areas (PAs) in the Pantanal and Uplands within the Brazilian boundaries of the Upper Paraguay River Basin (UPRB). **Type**: Split Protected Area classes in Integral Protection (IP) and Sustainable use (SU). **Type II**: Split the Protected Areas of Sustainable use in additional classes (APA = Environmental Protected Area with regulated sustainable use, RPPN = Private Protected Areas with restricted use, Park Road = buffer zone of restricted use along roads). Adapted from Tomas et al.[2] and Chaves & Silva[43]. Updated from[44–47] (direct links on Source).

Type	Type II	Region	Protected Area Name	Area	Year	Modification	Source
IP	IP	Uplands	PE Águas do Cuiabá	113.28	2002		https://uc.socioambiental.org/arp/2311
SU	APA	Uplands	APA das Cabeceiras do Rio Cuiabá	4734.11	1999		https://uc.socioambiental.org/arp/1348?order=field_data_documento_value&sort=asc
SU	APA	Uplands	APA Nascentes do Rio Paraguai	777.43	2006		https://uc.socioambiental.org/pt-br/arp/5114
SU	RPPN	Uplands	RPPN Fazenda São Luiz	2.04	1994		https://sistemas.icmbio.gov.br/simrppn/publico/detalhe/606/
IP	IP	Uplands	PN da Chapada dos Guimarães	330.00	1989		https://uc.socioambiental.org/pt-br/arp/599
SU	RPPN	Uplands	RPPN Mata Fria	0.10	2000		https://sistemas.icmbio.gov.br/simrppn/publico/detalhe/594/
SU	APA	Uplands	APA da Chapada dos Guimarães	2518.48	1995		https://uc.socioambiental.org/pt-br/arp/1142
SU	RPPN	Uplands	RPPN Hotel Mirante	0.20	2004		https://sistemas.icmbio.gov.br/simrppn/publico/detalhe/599/
SU	APA	Uplands	APA Aricá-açu	749.75	1999		https://www.icmbio.gov.br/parnaguimaraes/images/stories/downloads/encarte_1_p1_a_7.pdf
IP	IP	Uplands	PE Águas Quentes	14.87	1978		https://uc.socioambiental.org/pt-br/arp/1175
IP	IP	Uplands	MN Morro de Santo Antônio	2.58	2006		https://uc.socioambiental.org/pt-br/arp/4870
IP	IP	Uplands	PE Zé Bolo Flô	0.66	2000		https://uc.socioambiental.org/pt-br/arp/3546
IP	IP	Uplands	PE Mãe Bonifácia	0.77	2000		https://uc.socioambiental.org/pt-br/arp/3456
IP	IP	Uplands	EE da Serra das Araras	271.60	1982		https://www.icmbio.gov.br/portal/unidadesdeconservacao/biomas-brasileiros/cerrado/unidades-de-conservacao-cerrado/2067-esec-serra-das-araras
IP	IP	Uplands	PE Serra Santa Bárbara	1200.92	1997		https://uc.socioambiental.org/pt-br/arp/1345
SU	RPPN	Pantanal	RPPN Jubran	355.31	2002		https://sistemas.icmbio.gov.br/simrppn/publico/detalhe/597/
IP	IP	Pantanal	EE de Taiamã	112.00	1981		https://uc.socioambiental.org/arp/612
SU	RPPN	Pantanal	RPPN Estância Ecológica SESC - Pantanal	1079.96	1997		https://sistemas.icmbio.gov.br/simrppn/publico/detalhe/619/ https://sistemas.icmbio.gov.br/simrppn/publico/detalhe/620/ https://www.sescpantanal.com.br/hotel.aspx?s=12
SU	RPPN	Uplands	RPPN Parque Ecológico João Basso	36.25	1997		https://sistemas.icmbio.gov.br/simrppn/publico/detalhe/589/

IP	IP	Uplands	PE Dom Osório Stoffel	64.22	2002		https://uc.socioambiental.org/arp/3451
IP	IP	Pantanal	PE do Guirá	1102.26	2002		https://uc.socioambiental.org/pt-br/arp/3439
SU	RPPN	Pantanal	RPPN Estância Dorochê	265.18	1997		https://sistemas.icmbio.gov.br/simrppn/publico/detalhe/613/
IP	IP	Pantanal	PE Encontro das Águas	1089.60	2004		https://uc.socioambiental.org/pt-br/arp/4253
SU	RPPN	Pantanal	RPPN Poleiro Grande	165.30	1998		https://www.imasul.ms.gov.br/reserva-particular-do-patrimonio-natural-rppn/
SU	APA	Pantanal	APA Pontal dos Rios Itiquira e Correntes	2158.31	2003	2003, Revoked 2007	Chaves & Silva 2018
SU	APA	Pantanal	APA Pontal dos Rios Itiquira e Correntes	-2158.31	2007	2003, Revoked 2007	Chaves & Silva 2018
IP	IP	Pantanal	Monumento Natural Municipal da Serra do Pantanal	41.60	2005		https://sonora.ms.gov.br/v2/wp-content/uploads/2017/03/PLANO-DE-MANEJO_MONUMENTO-NATURAL-MUNICIPAL-DA-SERRA-DO-PANTANAL_OK.pdf
IP	IP	Pantanal	PN do Pantanal	1350.00	1981		https://uc.socioambiental.org/arp/600
SU	RPPN	Pantanal	RPPN Rumo ao Oeste	9.90	2005		https://www.imasul.ms.gov.br/reserva-particular-do-patrimonio-natural-rppn/
SU	RPPN	Pantanal	RPPN Fazenda Acurizal	132.00	2007		https://www.imasul.ms.gov.br/reserva-particular-do-patrimonio-natural-rppn/
SU	RPPN	Pantanal	RPPN Fazenda Penha	131.00	1997		https://www.imasul.ms.gov.br/reserva-particular-do-patrimonio-natural-rppn/
SU	RPPN	Pantanal	RPPN Engenheiro Eliezer Batista	133.23	2008		https://sistemas.icmbio.gov.br/simrppn/publico/detalhe/712/
SU	RPPN	Pantanal	RPPN Santa Cecília	88.41	1998	Additional area 2011	https://www.imasul.ms.gov.br/reserva-particular-do-patrimonio-natural-rppn/
SU	RPPN	Uplands	RPPN Cachoeiras do São Bento	30.37	2010		https://www.imasul.ms.gov.br/reserva-particular-do-patrimonio-natural-rppn/
IP	IP	Uplands	MN Serra do Bom Jardim	61.21	2003		http://www.wikiaves.com.br/wiki/areas:mona_serra_bom_jardim:inicio http://www.semagro.ms.gov.br/wp-content/uploads/2019/01/Encarte-1_Plano-de-Manejo-PENT.pdf
IP	IP	Uplands	PNM Templo dos Pilares	1.06	2003		http://www.semagro.ms.gov.br/wp-content/uploads/2019/01/Encarte-1_Plano-de-Manejo-PENT.pdf
IP	IP	Uplands	PE Nascentes do Rio Taquari	306.18	1999		https://www.imasul.ms.gov.br/gestao-de-unidades-de-conservacao/unidades-de-conservacao-estaduais/parque-estadual-nascentes-do-rio-taquari/
SU	Park Road	Pantanal	Área de Especial Interesse Turístico (AEIT) Estrada Parque Pantanal	68.00	1993		https://www.imasul.ms.gov.br/estrada-parque-do-pantanal-2/
SU	Park Road	Pantanal	Estrada-Parque Santo Anténio de Leverger – Porto de Fora – Baráo de Melgaco	44.70	2000		Chaves & Silva 2018 https://documentacao.socioambiental.org/ato_normativo/UC/2498_20170130_171941.pdf
SU	Park Road	Pantanal	Estrada-Parque Rodovia MT 370	39.23	2000		Chaves & Silva 2018
SU	RPPN	Pantanal	RPPN Arara Azul	2.04	2002		https://sistemas.icmbio.gov.br/simrppn/publico/detalhe/268/

SU	APA	Pantanal	APA da Baía Negra	54.21	2010	http://www.semagro.ms.gov.br/wp-content/uploads/2019/01/Encarte-1_Plano-de-Manejo-PENT.pdf
IP	IP	Pantanal	PNM de Piraputangas	13.00	2003	http://www.semagro.ms.gov.br/wp-content/uploads/2019/01/Encarte-1_Plano-de-Manejo-PENT.pdf
SU	RPPN	Pantanal	RPPN Paculândia	82.32	2002	https://www.imasul.ms.gov.br/reserva-particular-do-patrimonio-natural-rppn/
SU	RPPN	Pantanal	RPPN Fazenda Santa Helena	42.95	2000	https://sistemas.icmbio.gov.br/simrppn/publico/detalhe/622/
SU	RPPN	Pantanal	RPPN Fazendinha	96.16	1994	https://sistemas.icmbio.gov.br/simrppn/publico/detalhe/272/
SU	RPPN	Pantanal	RPPN Fazenda Alegria	11.35	2008	https://www.imasul.ms.gov.br/reserva-particular-do-patrimonio-natural-rppn/
SU	RPPN	Pantanal	RPPN Fazenda Nhumirim	8.63	1999	https://www.imasul.ms.gov.br/reserva-particular-do-patrimonio-natural-rppn/
IP	IP	Pantanal	PE do Pantanal do Rio Negro	783.03	2000	https://parquesnobrasil.org/arp/2933 http://www.servicos.ms.gov.br/imasuldownloads/PlanosdeManejo/planomanejoPEPRN.pdf
SU	RPPN	Pantanal	RPPN Pata da Onça (Faz. Santa Sophia)	73.87	1999	https://www.imasul.ms.gov.br/reserva-particular-do-patrimonio-natural-rppn/
SU	RPPN	Pantanal	RPPN Neivo Pires I and II	4.39	2001	https://www.imasul.ms.gov.br/reserva-particular-do-patrimonio-natural-rppn/
SU	RPPN	Pantanal	RPPN Estância Caiman	56.03	2004	https://sistemas.icmbio.gov.br/simrppn/publico/detalhe/253/
SU	RPPN	Pantanal	RPPN Fazenda Rio Negro	70.00	2001	https://www.imasul.ms.gov.br/reserva-particular-do-patrimonio-natural-rppn/
SU	APA	Uplands	APA Sete Quedas de Rio Verde	188.25	2005	https://www.rioverde.ms.gov.br/wp-content/uploads/2016/10/RESUMO-EXECUTIVO-APA-Sete-Quedas-do-Rio-Verde.pdf
SU	APA	Uplands	APA Rio Cênico Rotas Monçoeiras	154.40	2000	http://www.imasul.ms.gov.br/wp-content/uploads/2015/06/APA-Rio-C%C3%AAnico-Plano-Executivo.pdf
SU	RPPN	Uplands	RPPN Duas Pedras	1.53	2008	https://www.imasul.ms.gov.br/reserva-particular-do-patrimonio-natural-rppn/ https://www.imasul.ms.gov.br/wp-content/uploads/2019/08/Planilha-CNUC_para-o-site_atualiz.-Dez_-2018.pdf
SU	RPPN	Uplands	RPPN Cabeceira da Lagoa	4.31	2011	https://www.imasul.ms.gov.br/reserva-particular-do-patrimonio-natural-rppn/
SU	RPPN	Uplands	RPPN Vale do Bugio	0.82	2003	https://www.imasul.ms.gov.br/reserva-particular-do-patrimonio-natural-rppn/
SU	RPPN	Uplands	RPPN Fazenda Lageado	125.50	1990	https://www.imasul.ms.gov.br/reserva-particular-do-patrimonio-natural-rppn/
SU	APA	Uplands	APA Estrada Parque de Piraputanga	101.08	2000	https://www.imasul.ms.gov.br/wp-content/uploads/2019/08/Planilha-CNUC_para-o-site_atualiz.-Dez_-2018.pdf https://www.imasul.ms.gov.br/gestao-de-unidades-de-conservacao/unidades-de-conservacao-estaduais/estrada-parque-piraputanga/
SU	RPPN	Uplands	RPPN Cara da Onça	0.12	2009	https://www.imasul.ms.gov.br/wp-content/uploads/2019/08/Planilha-CNUC_para-o-site_atualiz.-Dez_-2018.pdf
IP	IP	Uplands	PN da Serra da Bodoquena	770.22	2000	https://www.icmbio.gov.br/portal/unidadesdeconservacao/biomas-brasileiros/cerrado/unidades-de-conservacao-cerrado/2082-parna-da-serra-da-bodoquena
SU	RPPN	Uplands	RPPN Fazenda Singapura	4.56	1994	https://sistemas.icmbio.gov.br/simrppn/publico/detalhe/251/
IP	IP	Uplands	MN da Gruta do Lago Azul	2.74	2001	https://www.imasul.ms.gov.br/wp-content/uploads/2019/08/Planilha-CNUC_para-o-site_atualiz.-Dez_-2018.pdf
SU	RPPN	Uplands	RPPN Rancho do Tucano	0.30	2011	https://www.imasul.ms.gov.br/reserva-particular-do-patrimonio-natural-rppn/
IP	IP	Uplands	MN do Rio Formoso	0.18	2003	https://www.imasul.ms.gov.br/wp-content/uploads/2019/08/Planilha-CNUC_para-o-site_atualiz.-Dez_-2018.pdf

SU	RPPN	Uplands	RPPN Fazenda São Pedro da Barra	0.88	2003	https://www.imasul.ms.gov.br/wp-content/uploads/2019/08/Planilha-CNUC_para-o-site_atualiz.-Dez_-2018.pdf
SU	RPPN	Uplands	RPPN Fazenda América	4.01	1994	https://sistemas.icmbio.gov.br/simrppn/publico/detalhe/263/
SU	RPPN	Uplands	RPPN Fazenda São Geraldo	6.42	1999	https://www.imasul.ms.gov.br/reserva-particular-do-patrimonio-natural-rppn/
SU	RPPN	Uplands	RPPN Cabeceira do Prata	3.08	1999	https://www.imasul.ms.gov.br/reserva-particular-do-patrimonio-natural-rppn/
SU	RPPN	Uplands	RPPN Buraco das Araras	0.29	2007	https://www.imasul.ms.gov.br/reserva-particular-do-patrimonio-natural-rppn/
SU	RPPN	Uplands	RPPN Xodó do Vô Ruy	4.88	2006	https://www.imasul.ms.gov.br/reserva-particular-do-patrimonio-natural-rppn/
SU	RPPN	Uplands	RPPN Fazenda Margarida	19.99	2000	https://sistemas.icmbio.gov.br/simrppn/publico/detalhe/266/
SU	Park Road	Uplands	Transpantaneira	74.29	1996	Chaves & Silva 2018

Tab.S4. Comparative ANOVAs considering the effect of years, location (project_region), and individuals (id) on fire occurrence within jaguar HRs during individual monitoring (monitoring time, HR in blue) or within the assumed home ranges (comparing all years, HR in red). The interaction between year and region exhibited the lowest residuals and the lowest AIC in both comparisons.

Models	Monitoring time (HR)				All years (HR)			
	residuals		*AIC*		*residuals*		*AIC*	
	df	*Mean Sq*	*df*	ΔAIC	*df*	*Mean Sq*	*df*	*ΔAIC*
*** interaction <- aov(fires1 ~ year : project_region, data = HR or HR**	**63**	**108.1**	**27**	**0**	**656**	**169**	**27**	**0**
one.way.year <- aov(fires1 ~ year, data = HR or HR)	78	415.8	12	108.9	752	460	12	549.5
one.way.local <- aov(fires1 ~ project_region, data = HR or HR)	82	561.1	8	132.0	761	586	8	1302.8
two.way <- aov(fires1 ~ year + project_region, data = HR or HR)	72	412.3	18	113.1	746	414	18	530.7
block_ID <- aov(fires1 ~ year + project_region + id, data = HR or HR)	71	414.2	19	114.2	745	414	19	532.7

Tab.S5. Estimates of burned areas in the Pantanal in 2020. Reference sources: (LASA[48], Libonati et al.[49]).

Region	Type	Burned Area 2020 (%)	Burned Area (ha)
APA Baía Negra	SU	74	45000
ESEC de Taiamã	IP	40	4700
PARNA do Pantanal Matogrossense	IP	100	136100
Parque Municipal de Piraputangas	IP	0	0
PE Encontro das Águas	IP	86	93300
PE do Guirá	IP	43	45300
PE do Pantanal do Rio Negro	IP	10	8200
RPPN Acurizal	SU	85	10400
RPPN Arara Azul	SU	90	1900
RPPN Dorochê	SU	84	22600
RPPN Estância Caiman	SU	0	0
RPPN Fazenda Nhumirim	SU	0	0
RPPN Fazenda Paculândia	SU	58	4800
RPPN Fazenda Rio Negro	SU	0	0
RPPN Fazenda Santa Cecília II	SU	29	2600
RPPN Fazenda Santa Sofia	SU	0	0
RPPN Fazendinha	SU	0	0
RPPN Jubran	SU	78	26300
RPPN Neivo Pires	SU	0	0
RPPN Penha	SU	100	12900
RPPN Pioneira do Rio Piquiri	SU	0	0
RPPN Poleiro Grande	SU	68	11200
RPPN Reserva Natural Eng. Eliezer Batista	SU	84	10600
RPPN Rumo ao Oeste	SU	60	600

RPPN Sesc Pantanal	SU	90	78800
TI Baía dos Guató	TI	94	18100
TI Cachoeirinha	TI	2	600
TI Guató	TI	61	900
TI Kadiwéu	TI	46	248000
TI Perigara	TI	86	9300
TI Taunay/Ipegue	TI	18	6000
TI Tereza Cristina	TI	86	25000
Fazenda Jatobazinho	Farms	89	300
Fazenda Morro Alegre	Farms	85	1200
Fazenda Porto Jofre	Farms	98	41500
Fazenda Santa Rosa	Farms	64	100
Fazenda Santa Tereza	Farms	75	44800
Fazenda São Bento	Farms	68	18800
Fazenda São Gonçalo	Farms	100	300
Fazenda Vale do Paraíso	Farms	81	200

Tab.S6. Model selection and home range output used for status classification in combination with variograms (ctmm)[50,51].

N	data id	DOF $_{area}$	τ_p (days)	τ_v (h)	duration (months)	model	status	Total points	start	end	HR (km²)	area CI (km²)	project	country
1	13	68.9	3.4	0.3	8.8	OUF_ani	R	5039	07-12-14	24-08-15	52.8	(41.1 - 66)	Taiama	Brazil
2	15	17.5	3.9	0.5	2.6	OUF_ani	R	1257	19-10-13	03-01-14	352.5	(206.95 - 536.09)	Oncafari	Brazil
3	18	26.0	4.7	0.4	4.6	OUF_ani	R	2305	29-11-14	13-04-15	126.8	(82.79 - 180.03)	Taiama	Brazil
4	19	53.6	0.9	0.3	2.5	OUF_ani	R	741	30-10-11	11-01-12	40.2	(30.18 - 51.68)	Oncafari	Brazil
5	22	22.0	10.0	0.3	8.5	OUF_ani	R	4709	11-09-14	21-05-15	114.0	(71.38 - 166.35)	Taiama	Brazil
6	25	102.5	1.6	0.2	35.3	OUF_ani	R	3074	22-10-12	30-08-15	46.2	(37.69 - 55.56)	Oncafari	Brazil
7	27	57.7	3.7	NA	39.3	OU_ani	R	559	21-09-10	24-11-13	51.2	(38.87 - 65.27)	Panthera2	Brazil
8	28	40.5	2.1	NA	3.3	OU_ani	R	205	08-07-10	13-10-10	33.2	(23.77 - 44.17)	Panthera1	Brazil
9	29	27.0	1.7	2.8	2.8	OUF_ani	R	67	08-07-11	30-09-11	43.3	(28.55 - 61.15)	Panthera1	Brazil
10	30	123.1	2.9	NA	13.6	OU_iso	R	581	13-10-11	19-11-12	60.8	(50.57 - 72.05)	Panthera2	Brazil
11	31	33.2	2.2	NA	3.5	OU ani	R	103	21-10-13	01-02-14	31.7	(21.84 - 43.33)	Panthera2	Brazil
12	32	92.8	1.3	NA	6.1	OU_ani	R	240	15-10-12	12-04-13	100.0	(80.7 - 121.36)	Panthera2	Brazil
13	33	26.6	2.9	NA	3.4	OU_ani	R	133	22-10-13	29-01-14	88.8	(58.33 - 125.62)	Panthera2	Brazil
14	41	61.0	3.8	0.3	8.6	OUF_ani	R	4951	05-12-14	17-08-15	25.5	(19.53 - 32.33)	Taiama	Brazil
15	52	6.5	3.6	0.3	0.9	OUF_ani	R	615	27-11-14	25-12-14	21.7	(8.34 - 41.33)	Taiama	Brazil
16	53	24.9	6.1	NA	37.9	OU_ani	R	299	20-09-10	13-10-13	348.6	(225.27 - 498.39)	Panthera2	Brazil
17	54	7.3	5.1	1.6	1.5	OUF_ani	R	128	12-07-10	26-08-10	36.1	(14.79 - 66.72)	Panthera1	Brazil
18	55	38.3	1.9	NA	4.2	OU_ani	R	141	26-06-11	28-10-11	91.8	(65.04 - 123.03)	Panthera2	Brazil
19	56	28.1	2.3	1.4	3.0	OUF_ani	R	109	08-07-11	04-10-11	72.6	(48.29 - 101.88)	Panthera1	Brazil
20	59	118.0	2.5	1.9	27.1	OUF_ani	R	434	15-10-11	23-12-13	241.3	(199.72 - 286.72)	Panthera2	Brazil
21	60	235.5	1.2	NA	12.8	OU_ani	R	705	10-10-12	23-10-13	88.4	(77.42 - 99.98)	Panthera2	Brazil
22	61	8.6	7.0	2.3	2.4	OUF_ani	R	109	18-06-13	28-08-13	343.9	(153.75 - 609.23)	Panthera2	Brazil
23	68	14.0	3.0	0.4	1.8	OUF_ani	R	996	01-11-11	23-12-11	242.9	(132.71 - 385.72)	Oncafari	Brazil
24	69	63.9	3.0	0.3	6.7	OUF_ani	R	3304	27-10-13	13-05-14	156.6	(120.55 - 197.23)	Oncafari	Brazil
25	79	42.9	2.8	0.4	4.3	OUF_ani	R	2202	19-04-15	25-08-15	68.9	(49.81 - 90.93)	Oncafari	Brazil
26	84	114.2	2.3	0.3	9.3	OUF_ani	R	4643	21-04-13	21-01-14	70.4	(58.05 - 83.84)	Oncafari	Brazil

27	86	26.4	3.0	0.3	3.0	OUF_ani	R	1324	22-10-13	17-01-14	166.3	(108.97 - 235.44)	Oncafari	Brazil
28	87	35.2	0.6	0.3	1.2	OUF_ani	R	398	15-05-12	18-06-12	37.4	(26.05 - 50.65)	Oncafari	Brazil
29	88	18.7	8.8	NA	6.6	OU_iso	R	1289	08-10-13	20-04-14	80.2	(48.1 - 120.48)	Taiama	Brazil
30	91	48.3	0.7	1.8	13.1	OUF_ani	R	85	01-01-11	24-01-12	66.8	(49.33 - 86.96)	Taiama	Brazil
31	92	81.6	0.2	NA	25.3	OU_ani	R	95	01-01-11	18-01-13	130.8	(103.97 - 160.67)	Taiama	Brazil
32	101	79.5	2.1	1.2	7.3	OUF_ani	R	404	26-08-15	29-03-16	302.1	(239.34 - 371.98)	RioNegro	Brazil
33	104	15.4	3.3	0.6	10.4	OUF_ani	R	134	22-08-15	23-06-16	481.7	(272.14 - 749.97)	RioNegro	Brazil
34	105	180.2	2.4	NA	16.1	OU_ani	R	2111	05-07-08	22-10-09	105.2	(90.43 - 121.15)	SaoBento	Brazil
35	106	48.2	1.0	1.5	25.9	OUF_ani	R	227	24-09-09	29-10-11	244.4	(180.32 - 318.01)	SaoBento	Brazil
36	107	19.9	3.8	0.8	4.5	OUF_ani	R	287	30-07-08	08-12-08	124.8	(76.11 - 185.43)	SaoBento	Brazil
37	108	72.3	1.3	0.3	3.5	OUF_ani	R	481	25-07-08	06-11-08	142.8	(111.77 - 177.54)	SaoBento	Brazil
38	109	69.6	0.6	NA	2.7	OU_ani	R	165	04-08-08	22-10-08	55.0	(42.86 - 68.68)	SaoBento	Brazil
39	110	53.9	1.1	NA	3.4	OU_ani	R	166	02-02-10	13-05-10	265.8	(199.62 - 341.33)	SaoBento	Brazil
40	111	104.4	4.5	NA	34.3	OU_ani	R	1757	17-07-08	26-04-11	58.4	(47.73 - 70.11)	SaoBento	Brazil
41	112	9.5	7.5	NA	3.0	OU_ani	R	202	16-07-08	13-10-08	119.1	(55.93 - 205.82)	SaoBento	Brazil
42	113	151.9	1.2	NA	11.5	OU_ani	R	707	11-08-08	15-07-09	40.6	(34.4 - 47.31)	SaoBento	Brazil
43	115	58.0	3.9	0.9	8.9	OUF_ani	R	952	20-06-08	10-03-09	123.8	(94.02 - 157.67)	SaoBento	Brazil
44	116	9.0	18.3	0.4	6.4	OUF_ani	R	3340	11-10-15	18-04-16	282.0	(129.18 - 493.57)	Taiama	Brazil
45	117	25.2	4.5	0.4	4.3	OUF_ani	R	2820	11-10-15	14-02-16	47.8	(30.95 - 68.13)	Taiama	Brazil
46	12	4.0	32.7	0.4	4.6	OUF_ani	NR	2681	05-12-14	18-04-15	619.7	(169.85 - 1355.38)	Taiama	Brazil
47	23	3.3	8.6	0.2	0.9	OUF_ani	NR	572	01-09-14	26-09-14	44.8	(10.23 - 104.34)	Taiama	Brazil
48	81	9.45*	52.7	0.4	20.0	OUF_ani	NR	10617	15-10-13	29-05-15	591.1	(276.34 - 1023.36)	Taiama	Brazil
49	102	4.6	12.2	1.1	1.9	OUF_ani	NR	151	29-03-16	23-05-16	567.4	(172.2 - 1193.86)	RioNegro	Brazil
50	51	47.6	3.6	1.4	6.5	OUF_ani	R	727	05-06-10	14-12-10	535.7	(394.46 - 698.22)	PantPy	Paraguay
51	74	98.6	2.6	NA	9.1	OU_ani	R	1300	09-08-05	06-05-06	75.4	(61.24 - 90.97)	PantPy	Paraguay
52	75	74.7	4.4	1.1	12.7	OUF_ani	R	1694	09-08-05	18-08-06	115.9	(91.13 - 143.64)	PantPy	Paraguay

N = individual id, data id = correspondent id in Morato et al. datapaper[40], DOF_{area} = effective number of range crossings, OUF_{ani} = anisotropic Ornstein-Uhlenbeck-F model, OUF_iso = isotropic Ornstein-Uhlenbeck-F model, R = resident jaguars, NR = non-resident jaguars. PantPy = Paraguayan Pantanal. Note that all non-resident jaguars had DOF area < 5, except for individual 81 (which had the largest amount of tracking locations in the dataset); however, observation of the variograms showed that this individual is a non-resident (see Fig.S21).

Supplementary References

1. Petry et al. Ecological Risk Assessment for the Paraguay River Basin. Argentina, Bolivia, Brazil and Paraguay. *Executive Summary*, 16 p. (TNC, WWF, 2012).
2. Tomas, W. M. *et al.* Sustainability Agenda for the Pantanal Wetland: Perspectives on a Collaborative Interface for Science, Policy, and Decision-Making. *Tropical Conservation Science* **12**, 1-30, (2019); https://doi.org/10.1177/1940082919872634.
3. Libonati, R. et al. Assessing the role of compound drought and heatwave events on unprecedented 2020 wildfires in the Pantanal. Environ. Res. Lett. 17, 015005 (2022).
4. Alho, C. J. R., Mamede, S. B., Benites, M., Andrade, B. S. & Sepúlveda, J. J. O. Threats to the biodiversity of the Brazilian Pantanal due to land use and occupation. Ambient. soc. 22, (2019).
5. Bergier, I. *et al.* Amazon rainforest modulation of water security in the Pantanal wetland. *Science of The Total Environment* **619–620**, 1116–1125 (2018).
6. Bergier, I. Effects of highland land-use over lowlands of the Brazilian Pantanal. *Science of The Total Environment* **463–464**, 1060–1066 (2013).
7. Alho, C. & Silva, J. Effects of Severe Floods and Droughts on Wildlife of the Pantanal Wetland (Brazil)—A Review. *Animals* **2**, 591–610 (2012).
8. Ioris, A., Irigaray, C. & Girard, P. Institutional responses to climate change: opportunities and barriers for adaptation in the Pantanal and the Upper Paraguay River Basin. *Climatic Change* **127**, 139–151 (2014).
9. Roque, F. O. *et al.* Upland habitat loss as a threat to Pantanal wetlands. *Conserv Biol* **30**, 1131–1134 (2016).
10. May Júnior, J. A. *et al.* Mercury content in the fur of jaguars (Panthera onca) from two areas under different levels of gold mining impact in the Brazilian Pantanal. *An. Acad. Bras. Ciênc.* **90**, 2129–2139 (2018).
11. Ely, P., Fantin-Cruz, I., Tritico, H. M., Girard, P. & Kaplan, D. Dam-Induced Hydrologic Alterations in the Rivers Feeding the Pantanal. *Front. Environ. Sci.* **0**, (2020).
12. Jardim, P. F.et al. Modeling Assessment of Large-Scale Hydrologic Alteration in South American Pantanal Due to Upstream Dam Operation. *Front. Environ. Sci.* **8**, (2020); https://doi.org/10.3389/fenvs.2020.567450 (2020).
13. Oliveira, M. D. et al. Further Development of Small Hydropower Facilities May Alter Nutrient Transport to the Pantanal Wetland of Brazil. *Front. Environ. Sci.* **8**, (2020); https://doi.org/10.3389/fenvs.2020.577793
14. Nogueira, F., Silva, E. C. E. & Junk, W. Mercury from gold minings in the Pantanal of Pocone (Mato Grosso, Brazil). *International Journal of Environmental Health Research* **7**, 181–192 (1997).
15. Bucher, E. H. & Huszar, P. C. Critical environmental costs of the Paraguay-Paraná waterway project in South America. *Ecological Economics* **15**, 3–9 (1995).
16. Gottgens, J. F. *et al.* The Paraguay-Paraná Hidrovia: Protecting the Pantanal with Lessons from the Past. *BioScience* **51**, 301–308 (2001).112.
17. Hamilton, S. K. Potential effects of a major navigation project (Paraguay–Paraná Hidrovía) on inundation in the Pantanal floodplains. *Regulated Rivers: Research & Management* **15**, 289–299 (1999).
18. Sousa Júnior et al. Nova Hidrovia Paraguai-Paraná: uma análise abrangente: análise de conjuntura e factibilidade política, econômica, social e ambiental da "nova" proposta da hidrovia Paraguai-Paraná (2019); https://mupan.org.br/_files/200001170-653776537a/20191021_Nova-Hidrovia-Paraguai-Parana_uma-analise-abrangente_e-book-3.pdf

19. MPF - Ministério Público Federal. Usinas hidrelétricas: MPF defende estudo na bacia hidrográfica do Pantanal Mato-Grossense para concessão de licença (2020)*;* http://www.mpf.mp.br/pgr/noticias-pgr/usinas-hidreletricas-mpf-defende-estudo-na-bacia-hidrografica-do-pantanal-mato-grossense-para-concessao-de-licenca
20. MPF/MS - Ministério Público Federal do Mato Grosso do Sul. Suspensa instalação de novas hidrelétricas no Pantanal. *Jusbrasil* (2013); https://mpf.jusbrasil.com.br/noticias/100039926/mpf-ms-suspensa-instalacao-de-novas-hidreletricas-no-pantanal
21. Habets, F., Molénat, J., Carluer, N., Douez, O. & Leenhardt, D. The cumulative impacts of small reservoirs on hydrology: A review. *Science of The Total Environment* **643**, 850–867 (2018).
22. Ioris, A. Rethinking Brazil's Pantanal Wetland: Beyond Narrow Development and Conservation Debates. *The Journal of Environment & Development* **22**, 239–260 (2013).
23. Odum, W. E. Environmental Degradation and the Tyranny of Small Decisions. *BioScience* **32**, 728–729 (1982).
24. Dias, A. M. S., Cook, C., Massara, R. L. & Paglia, A. P. Are Environmental Impact Assessments effectively addressing the biodiversity issues in Brazil? Environmental Impact Assessment Review 95, 106801 (2022).
25. MMA - Ministério do Meio Ambiente. *Livro Verde da Avaliação Ambiental Estratégica do Pantanal*. (2008).
26. ANA - Agência Nacional de Águas. Strategic action program for the integrated management of the Pantanal and the Upper Paraguay River Basin, *report,* Brasília, DF: ANA/GEF/PNUMA/OEA. 315 p (2004); https://arquivos.ana.gov.br/projetos/gefpantanal/PAE_Pantanal_EN.pdf
27. Neide, R. Comissão Externa destinada a acompanhar e promover a estratégia nacional para enfrentar as queimadas em biomas brasileiros – CEXQUEI, Bioma Pantanal, *Relatório*, 298 p, (Câmara dos Deputados, 2020); https://www.camara.leg.br/proposicoesWeb/prop_mostrarintegra?codteor=1949330&filename=REL+1/2020+CEXQUE
28. Azevedo-Santos, V. M. *et al.* Removing the abyss between conservation science and policy decisions in Brazil. *Biodivers Conserv* 26, 1745–1752 (2017).
29. Pardini, R. *et al.* COVID-19 pandemic as a learning path for grounding conservation policies in science. *Perspectives in Ecology and Conservation* (2021); doi:10.1016/j.pecon.2021.02.009.
30. INPE-Instituto Nacional de Pesquisas Espaciais. Monitoramento dos Focos Ativos por Estado, Região ou Bioma (Pantanal) – *(Programa Queimadas,* accessed 20 January 2021); http://queimadas.dgi.inpe.br/queimadas/portal-static/estatisticas_estados/
31. FIRMS, F. I. for R. M. S. FIRMS: Fire Information for Resource Management System. *Google Developers* (2020)*;* https://developers.google.com/earth-engine/datasets/catalog/FIRMS
32. MapBiomas - Projeto MapBiomas, Mapeamento das áreas queimadas no Brasil (Coleção 1), accessed in 19th April 2021: https://mapbiomas.org/en/colecoes-mapbiomas-1?cama_set_language=en , or https://code.earthengine.google.com/?scriptPath=users%2Fmapbiomas%2Fuser-toolkit%3Amapbiomas-user-toolkit-fire.js
33. CPRM/SGB - Serviço Geológico do Brasil. *HidroSeries, Um aplicativo para acesso simplificado aos dados hidrológicos do Sistema Nacional de Informações em Recursos Hídricos (SNIRH) e geração de series históricas* (Rede Hidrometereológica Nacional, accessed 31 October 2020); https://apps.cprm.gov.br/hidro-series/
34. ANA - Agência Nacional de Águas. *HIDROWEB*, version 3.1.1 (accessed 31 October 2020); http://www.snirh.gov.br/hidroweb/apresentacao
35. Projeto MapBiomas - *Coleção 6.0 e 5.0 da Série Anual de Mapas de Cobertura e Uso de Solo do Brasil* (2021), (accessed 15 September 2021).

36. MODIS6 - MODIS Collection 6 NRT Hotspot / Active Fire Detections MCD14DL (2020).
37. INMET, Instituto Nacional de Metereologia. (*Banco de Dados Meteorológicos,* accessed 31 October 2020); https://bdmep.inmet.gov.br/
38. CPTEC/INPE - Instituto Nacional de Pesquisas Espaciais. Clima Evolução - *Evolução Mensal e Sazonal das Chuvas* (Região 88), (accessed 31 December 2020);
39. MapBiomas Project – Mapping of the Water Surface of Brazil Collection 1, accessed in 19th April 2021; https://mapbiomas.org/en/colecoes-mapbiomas-1?cama_set_language=en
40. Morato, R. G. *et al.* Jaguar movement database: a GPS-based movement dataset of an apex predator in the Neotropics. *Ecology* **99**, 1691–1691 (2018).
41. Gorelick, N. *et al.* Google Earth Engine: Planetary-scale geospatial analysis for everyone. *Remote Sensing of Environment* **202**, 18–27 (2017).
42. IBGE – Instituto Brasileiro de Geografia e Estatística. SIDRA - Sistema IBGE de Recuperação Automática, *Statistical data*, Table 3939: Efetivo dos rebanhos, por tipo de rebanho (bovino) (2021); https://sidra.ibge.gov.br/tabela/3939
43. Chaves, J. V. B. & Silva, J. S. V. Evolução das unidades de conservação no Pantanal no período de 1998 a 2018, *Anais 7º Simpósio de Geotecnologias no Pantanal*, Jardim, MS, 676–685 (Embrapa Informática Agropecuária/INPE 2018).
44. MMA- Ministério do Meio Ambiente. Unidades de Conservação - Protected Areas. *Download de dados geográficos* (2020); http://mapas.mma.gov.br/i3geo/datadownload.htm
45. IMASUL - Instituto de Meio Ambiente de Mato Grosso do Sul. Plano de Manejo do Parque Estadual Nascentes do Rio Taquari (2019); https://www.imasul.ms.gov.br/gestao-de-unidades-de-conservacao/unidades-de-conservacao-estaduais/parque-estadual-nascentes-do-rio-taquari/
46. ICMBio- Instituto Chico Mendes de Conservação da Biodiversidade. Unidades de Conservação nos Biomas Brasileiros (2021);
47. ICMBio/SIMRPPN - Instituto Chico Mendes de Conservação da Biodiversidade/ Sistema Informatizado de monitoria de RPPN (2021); https://sistemas.icmbio.gov.br/simrppn/publico/
48. LASA - Laboratório de Aplicações de Satélites Ambientais. *Área queimada Pantanal* (Universidade Federal do Rio de Janeiro, Version 17/11/2020); https://lasa.ufrj.br/noticias/area-queimada-pantanal-2020/
49. Libonati, R., DaCamara, C. C., Peres, L. F., Carvalho, L. A. S. de & Garcia, L. C. Rescue Brazil's burning Pantanal wetlands. *Nature* **588**, 217–219 (2020).
50. Calabrese, J. M., Fleming, C. H. & Gurarie, E. ctmm: an R package for analyzing animal relocation data as a continuous-time stochastic process. *Methods Ecol Evol* **7**, 1124–1132 (2016).
51. Fleming, C. H. & Calabrese, J. M. A new kernel density estimator for accurate home-range and species-range area estimation. *Methods Ecol Evol* **8**, 571–579 (2017).
52. IBGE - Instituto Brasileiro de Geografia e Estatística. Biomas e sistema costeiro-marinho do Brasil: compatível com a escala 1:250 000 (2019).
53. Dinerstein, E. *et al.* An Ecoregion-Based Approach to Protecting Half the Terrestrial Realm. *BioScience* **67**, 534–545 (2017).
54. US Department of State Office of the Geographer. Global LSIB: Large Scale International Boundary Polygons, Simplified (2017), (accessed 10 January 2021); https://developers.google.com/earth-engine/datasets/catalog/USDOS_LSIB_SIMPLE_2017.
