# Supplementary material for: Wildfires disproportionately affected jaguars in the Pantanal
Source: Commun Biol. 2022 Oct 13;5:1028. doi: 10.1038/s42003-022-03937-1 (PMC9561719; doi:10.1038/s42003-022-03937-1)
Supplement: Supplementary file 3 — Reporting Summary [file 42003_2022_3937_MOESM3_ESM.pdf]

nature portfolio

Corresponding author(s): Alan Eduardo de Barros

Last updated by author(s): 2022-08-27

Reporting Summary

Nature Portfolio wishes to improve the reproducibility of the work that we publish. This form provides structure for consistency and transparency in reporting. For further information on Nature Portfolio policies, see our Editorial Policies and the Editorial Policy Checklist.

Statistics

For all statistical analyses, confirm that the following items are present in the figure legend, table legend, main text, or Methods section.

n/a	Confirmed	
☐	☒	The exact sample size (n) for each experimental group/condition, given as a discrete number and unit of measurement
☐	☒	A statement on whether measurements were taken from distinct samples or whether the same sample was measured repeatedly
☐	☒	The statistical test(s) used AND whether they are one- or two-sided<br>*Only common tests should be described solely by name; describe more complex techniques in the Methods section.*
☐	☒	A description of all covariates tested
☐	☒	A description of any assumptions or corrections, such as tests of normality and adjustment for multiple comparisons
☐	☒	A full description of the statistical parameters including central tendency (e.g. means) or other basic estimates (e.g. regression coefficient) AND variation (e.g. standard deviation) or associated estimates of uncertainty (e.g. confidence intervals)
☒	☐	For null hypothesis testing, the test statistic (e.g. F, t, r) with confidence intervals, effect sizes, degrees of freedom and P value noted<br>*Give P values as exact values whenever suitable.*
☒	☐	For Bayesian analysis, information on the choice of priors and Markov chain Monte Carlo settings
☒	☐	For hierarchical and complex designs, identification of the appropriate level for tests and full reporting of outcomes
☐	☒	Estimates of effect sizes (e.g. Cohen's d, Pearson's r), indicating how they were calculated

Our web collection on statistics for biologists contains articles on many of the points above.

Software and code

Policy information about availability of computer code

Data collection

To assess the annual impact of fires (2005–2020, references 42,43) on jaguars, we used two main sources of data, as follows:
a) published estimates of jaguar abundance for its entire geographic range based on spatial predictions of density and distribution (reference 36) and
b) home range (HRs) areas estimated for 48 resident jaguars monitored between 2005 and 2016 in the Pantanal (reference 44).

Original jaguar movement data (reference 44) associated with this publication are available at https://doi.org/10.5061/dryad.2dh0223 (Dryad Digital Repository).
We provided raw and processed data, and related R codes at figshare: https://doi.org/10.6084/m9.figshare.17698595.v1

We used Google Earth Engine (GEE) computing platform through the Code Editor (a web-based IDE for writing and running scripts) to run geospatial analysis. We worked with JavaScript GEE client library freely available for non commercial research.
The majority of GIS data was acquired through GEE (e.g. landscape (reference 65), fire (references 42,43), Protected Areas (reference 97), country boundaries (reference 128)). And in the cases were it did not occur (such as boundaries for the Brazilian Protected Areas, references 95,96) or the raster for jaguar densities (reference 36) we included the layers as assets in our GEE code, or discussed independently such as hydrological and related data (references 52- 54, 116, 117).
A Google Earth Engine example from year 2020 (Main Code) shows datasets and assets used.
https://code.earthengine.google.com/f0ae619db0404f606562d33290416277

Original data with jaguar abundance estimates (reference 36, Jędrzejewski, W. et al 2018) was acquired through contact and with permission of the main authors.

Data analysis

- Jaguar data cleaning and preparation for temporal order and duplicates were performed in R (version 4.0.2), using amt (0.1.2) and ctmm

(0.5.10) packages.
- Autocorrelated kernel density estimator (AKDE) Home Ranges (HR) were calculated using ctmm (0.5.10).
- We followed J. Fieberg and J. Signer (amt package, reference 134) scripts for cleaning and preparing the basic movement data.
- The analyses of the jaguar home range followed Fleming et al. (ctmm package, references 131,132).
- Jaguar abundance estimates, home ranges, country boundaries and Protected Areas were all reclassified using the raster package (version 3.3-13) in the same grid alignment and resolution as the fire raster images.
- In brief, the impact of fire was assessed by multiplying the other layers by the fire occurrence raster. (See detailed explanation, and further particularities of each case, in the methods section).
R scripts with raster operations accounting for fire impacts on areas, jaguar abundances and home ranges are available at https://doi.org/10.6084/m9.figshare.17698595.v1 .

Program and packages:
R version 4.0.2 (2020-06-22) -- "Taking Off Again"
Copyright (C) 2020 The R Foundation for Statistical Computing
Platform: x86_64-w64-mingw32/x64 (64-bit)
attached base packages:
[1] stats graphics grDevices utils datasets methods base

other attached packages:
[1] DescTools_0.99.41 ggridges_0.5.2 ggpubr_0.4.0 ctmm_0.5.10 ezknitr_0.6 knitr_1.29 caTools_1.18.0
[8] circular_0.4-93 here_0.1 stringr_1.4.0 purrr_0.3.4 tidyr_1.1.1 tidyverse_1.3.0 forcats_0.5.0
[15] tibble_3.0.3 lubridate_1.7.9 readr_1.3.1 dplyr_1.0.1 RCurl_1.98-1.2 ggplot2_3.3.2 leaflet_2.0.3
[22] lattice_0.20-41 rgl_0.100.54 colorspace_1.4-1 maptools_1.0-1 amt_0.1.2 adehabitatLT_0.3.25 CircStats_0.2-6
[29] boot_1.3-25 MASS_7.3-51.6 adehabitatMA_0.3.14 ade4_1.7-15 move_4.0.4 rgdal_1.5-12 raster_3.3-13
[36] sp_1.4-2 geosphere_1.5-10 install.load_1.2.3

loaded via a namespace (and not attached):
[1] ggsignif_0.6.0 class_7.3-17 ellipsis_0.3.1 rio_0.5.16 rprojroot_1.3-2 fs_1.5.0
[7] gld_2.6.2 rstudioapi_0.11 fansi_0.4.1 mvtnorm_1.1-1 xml2_1.3.2 codetools_0.2-16
[13] splines_4.0.2 rootSolve_1.8.2.1 jsonlite_1.7.0 broom_0.7.10 dbplyr_1.4.4 shiny_1.5.0
[19] compiler_4.0.2 httr_1.4.2 backports_1.1.7 assertthat_0.2.1 Matrix_1.2-18 fastmap_1.0.1
[25] cli_2.0.2 later_1.1.0.1 htmltools_0.5.0 tools_4.0.2 lmom_2.8 gtable_0.3.0
[31] glue_1.4.1 fastmatch_1.1-0 Rcpp_1.0.5 carData_3.0-4 cellranger_1.1.0 vctrs_0.3.2
[37] crosstalk_1.1.0.1 xfun_0.16 openxlsx_4.2.3 rvest_0.3.6 mime_0.9 miniUI_0.1.1.1
[43] lifecycle_0.2.0 rstatix_0.6.0 scales_1.1.1 hms_0.5.3 promises_1.1.1 parallel_4.0.2
[49] expm_0.999-5 Exact_2.1 curl_4.3 memoise_1.1.0 stringi_1.4.6 e1071_1.7-3
[55] checkmate_2.0.0 zip_2.0.4 manipulateWidget_0.10.1 rlang_0.4.7 pkgconfig_2.0.3 bitops_1.0-6
[61] htmlwidgets_1.5.1 tidyselect_1.1.0 plyr_1.8.6 magrittr_1.5 R6_2.4.1 generics_0.0.2
[67] DBI_1.1.0 pillar_1.4.6 haven_2.3.1 foreign_0.8-80 withr_2.2.0 survival_3.1-12
[73] abind_1.4-5 modelr_0.1.8 crayon_1.3.4 car_3.0-10 utf8_1.1.4 grid_4.0.2
[79] readxl_1.3.1 data.table_1.13.0 blob_1.2.1 reprex_0.3.0 digest_0.6.25 webshot_0.5.2
[85] xtable_1.8-4 httpuv_1.5.4 munsell_0.5.0

We used Google Earth Engine computing platform through the Code Editor (a web-based ID platform for writing and running scripts) to run geospatial analysis.
Google Earth Engine example from 2020 (Main Code)
https://code.earthengine.google.com/f0ae619db0404f606562d33290416277

The same code was applied filtering fire to the other years (2001 to 2019).

For manuscripts utilizing custom algorithms or software that are central to the research but not yet described in published literature, software must be made available to editors and reviewers. We strongly encourage code deposition in a community repository (e.g. GitHub). See the Nature Portfolio guidelines for submitting code & software for further information.

Data

Policy information about availability of data

All manuscripts must include a data availability statement. This statement should provide the following information, where applicable:
- Accession codes, unique identifiers, or web links for publicly available datasets
- A description of any restrictions on data availability
- For clinical datasets or third party data, please ensure that the statement adheres to our policy

Original data associated with this publication, and our raw and processed data are available in the manuscript through the same links provided in the items above. Original data with jaguar adjusted densities estimates should be acquired directly with the main authors of reference 36 (Jędrzejewski, W. et al 2018), published data used with permission here.

Field-specific reporting

Please select the one below that is the best fit for your research. If you are not sure, read the appropriate sections before making your selection.

☐ Life sciences ☐ Behavioural & social sciences ☒ Ecological, evolutionary & environmental sciences

For a reference copy of the document with all sections, see nature.com/documents/nr-reporting-summary-flat.pdf

All studies must disclose on these points even when the disclosure is negative.

Study description	We addressed how fire has impacted the population sizes, home ranges, and priority areas for conservation of the jaguar.<br>We compared remote sensing data on fires that occurred in the last 16 years (2005-2020) in the Pantanal to investigate temporal trends of fire affecting (I) the number of jaguars, (II) the proportion and extent of areas selected as home ranges (HRs) by jaguars, and (III) the proportion and extent of legally protected areas (PAs) within the HR of individual jaguars. The figures provide an immediate visual perception of the impact (in percentage or absolute numbers). And in the text and table we showed effect factors considering distance of 2020 fire impacts in relation to the median (or mean) of fire in the previous years. As well as the connection of percentages of fire with the extent of the area affected and the intensity of fires.
Research sample	Overall impact in the Pantanal<br>As a spatial limit of the Pantanal, we adopted a merged image of the legal boundaries of the Brazilian Pantanal biome and Pantanal Ecoregion within the UPRB, totalling 160,426 km2. We calculated fire occurrences separately within each country's boundaries. The Pantanal area within Brazil corresponded to 150,893 km2 (150,355 km2 of the legal biome merged with additional Pantanal ecoregion areas within Brazil). The Pantanal ecoregion corresponded to 26,399 km2 within Bolivia and 1,970 km2 within Paraguay. Vectors for countries, ecoregion, and PAs boundaries were rasterized and resampled to match the 1-km resolution and then reclassified using GEE and the raster package from R statistical software.<br><br>1) Proxy for the number of jaguars affected by fires in the Pantanal<br>We used jaguar adjusted densities estimates (reference 36) occurring in the pixels reached by fires (references 42,43) as a proxy for the number of jaguars affected (e.g., potentially displaced, injured, or killed by fires (reference 40)) in 2020 and the previous 15 years.<br><br>2) Jaguar home range estimates<br>We used published data (reference 44) to estimate jaguar home ranges and evaluate the impact of fire on home ranges (HRs) during 2020 and the previous 15 years. We gathered GPS data on the movement of 56 individual jaguars tracked at seven sites in the Brazilian, Paraguayan, and Bolivian Pantanal. From these data, we used 48 individuals classified as residents. We excluded individuals with insufficient data or classified as non-residents (see SI).<br><br>We used jaguar adjusted density estimates (reference 36) in areas overlapping with the occurrence of fire gauges as a proxy for the number of animals potentially displaced, injured, or killed by fires (reference 45). Home-range areas were estimated from GPS tracking data (reference 44) of 45 jaguar individuals tracked in the Brazilian Pantanal and three in the Paraguayan and Bolivian Pantanal between 2005 and 2016. We selected only individuals whose HRs could be assumed as stable areas, capable of maintaining a resident animal or likely to be occupied by a new individual if conditions were kept similar.
Sampling strategy	Overall impact in the Pantanal<br>We evaluated the impact of fire in the Pantanal by overlapping raster images of the annual occurrence of fires and the Pantanal extent within each country. We reclassified the Pantanal boundaries so that the sum of the cell values was 1 and then multiplied these values by the raster of fire occurrence. This multiplication resulted in a distribution of the occurrence of fires, with the sum of these cells corresponding to an estimated proportion of the impact of fire in the Pantanal of each country. The mean (or median) annual fire intensity was calculated based on the pixels' mean (or median) values.<br>A similar process of resampling and reclassifying raster images was applied to evaluate the impact of fire on the PAs of the Pantanal. First, we calculated the extent of PAs in the Pantanal. Second, we calculated the extent of PAs impacted by fires — i.e., the probability of fire occurrence per pixel based on the multiplication of the Pantanal PAs raster by the fire occurrence raster. Then, we calculated the ratio between the PAs impacted by fire and the total extent of the Pantanal PAs in each country.<br><br>1) Proxy for the number of jaguars affected by fires in the Pantanal<br>We clipped the raster image output from Jędrzejewski et al. (reference36) containing jaguar abundance estimates with the Pantanal polygon masks of each country and adjusted the resolution to 1 km. As the original information corresponded to the estimated number of jaguars per 100 km2, we converted this information to a 1-km resolution by dividing the cells by 100, thus obtaining the number of jaguars per 1 km2. Therefore, the sum of the pixels corresponded to a proxy for the total number of jaguars within the boundary of the Pantanal area to be assessed (for Brazil, Bolivia, Paraguay, or the entire Pantanal). Next, we selected the pixels of jaguar density estimates overlapping with the occurrence of fire. Thus, the sum of the pixels with fire records corresponded to a proxy for the estimated number of individuals impacted by fire in the Pantanal in each country. Finally, we calculated the correspondent percentages of jaguars impacted by the fire.<br><br>2) Jaguar home range estimates affected by fire<br>Individual residency status was evaluated by analysing the asymptotic behaviour of semi-variograms (SI_Fig.S6, SI_Tab.S4) and complementary statistics, such as the estimated number of range crossings (Narea or DOFarea), with the continuous-time time movement modelling (ctmm) R package (references 40,131 - 133) . Individuals were classified as residents if they inhabited the

home-range area during the monitoring period, had DOFarea > 5 , or obtained an asymptote in their semi-variogram (references 40,131 - 133) (see SI). The minimum sampling period used was 27 days, and the maximum was 591 days (SI).
We calculated individual jaguar home ranges as indicative of areas selected as home ranges (HRs) using the Autocorrelated Kernel Density Estimator (AKDE), from the ctmm R package and the same grid alignment and resolution as the fire raster images. From each AKDE, we calculated the probability mass function, an indicator of the intensity of jaguar space use within the AKDE-derived raster images, and multiplied this value by the raster images of fire occurrence. The sum of the resulting probabilities at each pixel meant the proportion of individual jaguar HRs impacted by the fire. The annual fire intensities within HRs were calculated by averaging the fire intensity values recorded at each pixel. Lastly, we calculated the frequency distribution of jaguars in PAs, i.e., the extent of HRs included in PAs. Then we estimated the extent of HRs containing PAs with fire occurrence. To do so, we first multiplied the estimated probability mass function of each jaguar (corresponding to the jaguars' AKDE) by the occurrence of PAs. Next, we multiplied these two layers by the raster images of fire occurrence. These analyses (Figs. 1,2,3) consisted of comparing the impact of fire in all HRs (n = 48) over time (2005–2020).

Data collection

Original jaguar movement data associated with this publication have been published in 2018 (reference 44, Morato et al. 2018) and is freely available at https://doi.org/10.5061/dryad.2dh0223 (Dryad Digital Repository). This published work compiled movement data of 117 jaguars distributed in 7 countries of the species range. We used only data from the Pantanal, using the procedures described above to select only the resident individual.

Jaguar abundance estimates used in this study have been published by Jędrzejewski et al. 2018 (reference 36). In their study, Jędrzejewski et al. population estimates were derived from 80 studies of camera traps spread across the jaguar distribution between 2002 to 2014 (2005 for the Pantanal). Population density and probability of occurrence were then modelled by the authors as response variables to environmental covariates, such as net primary productivity. Finally, to adjust the estimates to the actual jaguar range, the authors multiplied the population density estimate by the probability of occurrence estimate.
Here, we used this adjusted abundance estimates (information that was clipped for the whole Pantanal).

Fire and other GIS data were collected from the sources described above and in the references.

Timing and spatial scale

The main proposal of the study was to evaluate the impacts of fire during 2020 and the previous 15 years. This started by getting an estimate of the overall impact of fire in the Pantanal biome during these years, but most importantly, our goal was to identify the impact of fire on jaguars in the Pantanal.

Fire: We gathered through GEE readily available data for fire occurrence for 2020 and for each of the previous 15 years.
Jaguar data: we used two main sources of data:
a) published estimates of jaguar abundance (based on year 2005 for the Pantanal, reference 36)
b) home range (HRs) areas estimated from movement data for 48 resident jaguars monitored between 2005 and 2016 in the Pantanal (reference 44).
We assumed the jaguar data as constants across years , in both cases. For a) this was the rationale adopted by the authors (reference 36). And for B) we assumed the areas selected as home range as stable areas, capable of maintaining a resident animal or likely to be occupied by a new individual if conditions were kept similar (2nd order habitat selection, reference 41).

We also estimated the real impact of fire in the years the animals were monitored. But in such case the reach of the results would be limited (see SI). Furthermore, the interaction between the place (project region) and time (year) were the most plausible model to explain occurrence of fires in both cases (considering just the GPS monitoring time, or assuming areas selected as home ranges as stable areas, capable of maintaining the monitored or other resident individual; see SI). Thus, the approach we adopted was a better way to take advantage of the data and compare the impact of fire on jaguars in 2020 and in the previous 16 years.

Data exclusions

We excluded non-resident individuals (as a pre-established criteria) because our goal was to identify the impact of fire on areas selected as home ranges, assuming them as stable areas, capable of maintaining the monitored or other resident individual. Non-resident individuals may often be in the search of better places, and consequently staying longer in non optimal habitat. Although the impact of fire on non-resident jaguars, still an impact over the species, we concluded that for our design and assumptions, we should focus only on resident jaguars.

Reproducibility

If the same jaguar data, GIS bases, scale and scripts were used the results should be the same.
The resampling of raster images may suffer slight changes depending on which sources are used to define the grid, but that should not affect the results.
The use of other sources with a finer or rougher scale, or with additional filtering processes for fire may cause some changes in the estimates of occurrence of fire or areas burned. However; we believe that even if absolute values for fire (increase or diminish a little) the results should be similar, as fire datasets are strongly correlated based on a quick exploration (see Supplementary Information).

Randomization

Fire occurrence data was grouped for year, with 1 Km resolution. Totals were calculated to the Pantanal and by (Pantanal area in each) country. Jaguar abundance estimates were resampled to 1 Km resolution and calculated for the Pantanal and by (Pantanal area in each) country. This information was overlapped with fire occurrence, and the impact of fire was then calculated for the Pantanal and by (Pantanal area in each) country. Home ranges were estimated for individuals, rasterized and resampled to 1 Km resolution and overlapped with fire rasters. The impact of fire was then calculated for the Pantanal and by (Pantanal area in each) country.
The main proposal of this study was the comparison of the fire impacts in 2020 with the 16 previous years, whose magnitude was so greater, that the comparison could be done even using basic descriptive statistics.

Blinding

All the data used came from published sources. Thus, we did not use any blinding scheme for data acquisition. We believed that, in [illegible]

the context of our objectives, the comparison of the impact of fires across the years was sufficient. Some blinding or control scheme could be used if our questions meant to compare jaguar responses in the occurrence, absence or distinct gradient of fire. But that would be more applicable to another scale, for instance in 3rd order resource selection studies, were areas used, indicated by the GPS points could be contrasted with random points. Indeed, a similar idea could be applicable to a 2 order selection (home ranges), but that would demand much more data and individuals, and that individuals should be monitored consistently over long periods of time to allow a case control comparison with fire as a predictor variable.

Did the study involve field work? ☐ Yes ☒ No

Reporting for specific materials, systems and methods

We require information from authors about some types of materials, experimental systems and methods used in many studies. Here, indicate whether each material, system or method listed is relevant to your study. If you are not sure if a list item applies to your research, read the appropriate section before selecting a response.

Materials & experimental systems

n/a	Involved in the study
☒	☐ Antibodies
☒	☐ Eukaryotic cell lines
☒	☐ Palaeontology and archaeology
☐	☒ Animals and other organisms
☒	☐ Human research participants
☒	☐ Clinical data
☒	☐ Dual use research of concern

Methods

n/a	Involved in the study
☒	☐ ChIP-seq
☒	☐ Flow cytometry
☒	☐ MRI-based neuroimaging

Animals and other organisms

Policy information about studies involving animals; ARRIVE guidelines recommended for reporting animal research

Laboratory animals	Not applicable
Wild animals	Used data from GPS tracking data of wild jaguars in the Pantanal was downloaded from available/previously published data (reference 44, https://doi.org/10.5061/dryad.2dh0223 (Dryad Digital Repository)).
Field-collected samples	Not applicable
Ethics oversight	Since we used previously published data all the Ethical approval related with the data have been applied to anterior studies.

Note that full information on the approval of the study protocol must also be provided in the manuscript.
